# Mind, rationality, and cognition: An interdisciplinary debate

**DOI:** 10.3758/s13423-017-1333-5

**Published:** 2017-07-25

**Authors:** Nick Chater, Teppo Felin, David C. Funder, Gerd Gigerenzer, Jan J. Koenderink, Joachim I. Krueger, Denis Noble, Samuel A. Nordli, Mike Oaksford, Barry Schwartz, Keith E. Stanovich, Peter M. Todd

**Affiliations:** 1 Warwick Business School, University of Warwick, Coventry, UK; 2 Saïd Business School, University of Oxford, Oxford, UK; 3 Psychology, University of California, Riverside, CA USA; 4 Adaptive Behavior and Cognition, Max Planck Institute for Human Development, Berlin, Germany; 5 Experimental Psychology, University of Leuven, Leuven, Belgium; 6 Cognitive, Linguistic, and Psychological Sciences, Brown University, Providence, RI USA; 7 Physiology, Anatomy, and Genetics, University of Oxford, Oxford, UK; 8 Psychological and Brain Sciences, Indiana University, Bloomington, IN USA; 9 Psychological Sciences, Birkbeck College, University of London, London, UK; 10 Psychology, Swarthmore College, Swarthmore, PA USA; 11 Psychology, University of California, Berkeley, CA USA; 12 Applied Psychology and Human Development, University of Toronto, Toronto, Ontario Canada

**Abstract** This article features an interdisciplinary debate and dialogue about the nature of mind, perception, and rationality. Scholars from a range of disciplines—cognitive science, applied and experimental psychology, behavioral economics, and biology—offer critiques and commentaries of a target article by Felin, Koenderink, and Krueger (2017): “Rationality, Perception, and the All-Seeing Eye,” *Psychonomic Bulletin & Review*. The commentaries raise a number of criticisms and issues concerning rationality and the all-seeing-eye argument, including the nature of judgment and reasoning, biases versus heuristics, organism–environment relations, perception and situational construal, equilibrium analysis in economics, efficient markets, and the nature of empirical observation and the scientific method. The debated topics have far-reaching consequences for the rationality literature specifically, as well as for the cognitive, psychological, and economic sciences more broadly. The commentaries are followed by a response from the authors of the target article. Their response is organized around three central issues: (1) the problem of cues; (2) what is the question?; and (3) equilibria, $500 bills, and the axioms of rationality.

**Keywords** Rationality, Perception, Cognition

**Introduction**

The cognitive sciences, psychology, and economics are intimately linked in their interest in rationality. Foundational to most conceptions of rationality, judgment, and reasoning is a particular view of perception. For example, Herbert Simon’s (1955, 1956) pioneering work on the boundedness of rationality—as an alternative to the omniscience of agents in economics—builds on this view of perception and vision. The subsequent cognitive and behavioral revolution in psychology and economics, and particularly the work of Kahneman and Tversky, further emphasized these perceptual foundations (see Kahneman, 2003, 2011). Simon’s and Kahneman’s research on bounded rationality has influenced and reverberated across cognitive and decision science, psychology, computer science, law, and economics (e.g., Camerer, 1998, 1999; Conlisk, 1996; Evans, 2008; Gershman, Horvitz, & Tenenbaum, 2015; Hills et al., 2015; Jolls, Sunstein, & Thaler,^1998^; Jones, 1999; Korobkin, 2015; Luan, Schooler, & Gigerenzer, 2014; Payne, Bettman, & Johnson, 1992; Puranam, Stieglitz, Osman, & Pillutla, 2015; Simon, 1980, 1990; Todd & Gigerenzer, 2003; Williamson, 1985).

Felin, Koenderink, and Krueger (2017; henceforth “Felin et al.”) argue that much of this work—which has challenged the assumption of agent omniscience—builds on a theoretically problematic and empirically flawed conception of rationality and perception, what they call an “all-seeing eye.” Felin et al. use familiar visual tasks and experiments as examples and show how assuming this all-seeing eye has led to wrong interpretations of not just rationality, but human nature and mind as well. Felin et al. suggest an alternative view of perception, drawing on biology, psychology, and the vision sciences. The authors also discuss some ways forward, by focusing on the multifarious nature of both perception and rationality.

The present article features commentaries on the target article by Felin et al. The commentaries are written by scholars from varied disciplines, including cognitive science, psychology, behavioral economics, biology, and physiology. Among the issues raised are matters such as the nature of judgment and reasoning, biases versus heuristics, organism–environment relations, perception and situational construal, equilibrium analysis in economics, efficient markets, and the nature of empirical observation and the scientific method. The commentators debate, critique, and discuss central aspects of rationality and the all-seeing-eye argument. The tones of the different commentaries range from severely critical to complimentary and supportive. Overall, the commentaries can be seen as a debate that vets many of the key issues and assumptions at the forefront of the rationality, perception, and cognition literatures.

By way of background, the commentators have done pioneering work in an array of areas related to rationality, reasoning, and cognition. For example, Nick Chater and Mike Oaksford (2006) have made high-profile contributions to our understanding of Bayesian rationality, and more recently to our understanding of reasoning and mental representation (Chater & Oaksford, 2013). Kenrick and Funder, (1988) is a central contributor to the person–situation debate in psychology, and more recently has developed a model of situational construal and perception (Funder, 2016; Rauthmann et al., 2014). Gerd Gigerenzer is a founding father of the heuristics literature, which has questioned existing understandings of cognitive biases (Gigerenzer, Todd, & the ABC Group, 1999). Related to this, he has also pioneered models of ecological rationality (e.g., Goldstein & Gigerenzer, 2002; Todd & Gigerenzer, 2012). Denis Noble’s (2008) work has focused on epigenetics, evolution and developmental biology, and physiology, and more recently on the idea of biological relativism (Noble, 2016). Keith Stanovich’s research has focused on dual-process models of rationality and mind (e.g., Evans & Stanovich, 2013) and individual differences in reasoning (Stanovich, 1999), and he has recently developed a so-called “rationality quotient” to test rationality and reasoning in humans (Stanovich, West, & Toplak, 2016). Barry Schwartz (2004) has written extensively about decision making, and recently has asked questions about the nature of rationality (Schwartz, 2015). Finally, Peter Todd has done pioneering work on heuristics and adaptive or ecological rationality with Gigerenzer, along with developing general models of rationality and search (e.g., Hills et al., 2015; Todd & Brighton, 2016; Todd & Gigerenzer, 2012).

It is worth nothing that the literatures on rationality cognition, and mind are not the only ones infused with perception-related issues and assumptions—so are the rest of the sciences. Indeed, as noted by Gigerenzer and Selten, “visions of rationality do not respect disciplinary boundaries” (2001, p.1). For example, theories of consciousness and the philosophy of mind inevitably anchor on particular views of perception (e.g., Block, 2014; Chalmers, 1996; Feyerabend, 1975; Noë, 2004), as do theories of rationality and perception (Siegel, 2017) and theories of aesthetics and art (Grootenboer, 2013; Hyman, 2006). All-seeing assumptions about perception and observation are also readily evident in equilibrium models in economics (Frank & Bernanke, 2003; Frydman & Phelps, 2001; Muth, 1961). Arguably, perception is at the very center of science itself; after all, theories are empirically tested through some manner of perceptual or observational means, in which varied scientific tools and perception-enhancing instruments are utilized to make observations and gather evidence (cf. Bell, 1990). Therefore, this discussion also has inevitable reverberations and links with various “-isms” in the philosophy of science, including different forms of realism, objectivism, idealism, and relativism (Dreyfus & Taylor, 2015; Haack, 2011; Van Fraassen, 2008).

Overall, our hope is that this debate will contribute to an interdisciplinary awareness and discussion about the nature of perception, as it relates to rationality, mind, judgment cognition, and beyond. Each of the seven commentaries serves as a subsection of this article. The commentaries can be found below (ordered alphabetically by first author), after which the target article authors respond to some of the central issues raised by the commentaries.

**Functional and equilibrium explanation: Two roles for rationality in the cognitive and social sciences**

Nick Chater, Mike Oaksford

Felin, Koenderink, and Krueger (2017) provide a wide-ranging examination of some of the hidden assumptions in the rational explanation of perception, judgment and decision making, and economics. This commentary argues that researchers across the biological and social sciences are right to seek rational explanation, and that the usefulness of such explanation need not depend on the assumption of an “all-seeing eye,” as Felin et al. suppose. In particular, their article distinguished between two types of rational explanation: equilibrium explanations that depend on coherence relations between beliefs, preferences, and actions (the style of explanation prevalent in economics), and functional explanations that optimize an externally defined information-processing problem (prevalent in explanations of perception). Felin et al.’s concerns about the “all-seeing eye” apply only to the latter category. But we argue that, despite these concerns, such explanations are of vital importance and are no more problematic than the functional explanation that is ubiquitous in biology.

Rational explanations of thought and behavior are central to our common sense understanding of each other’s behavior (e.g., Bratman, 1987; Fodor, 1987), are fundamental to explanation in economics and the social sciences (Binmore, 2008), and underpin information-processing accounts of cognition (Anderson, 1990, 1991; Anderson & Schooler, 1991; Oaksford & Chater, 1998, 2007; Tenenbaum, Griffiths, & Kemp, 2006; Tenenbaum, Kemp, Griffiths, & Goodman, 2011). Felin et al. identify what they see as a crucial hidden assumption underlying much rational explanation, focusing particularly on the domains of perception and of judgment and decision making. They argue that “the cognitive and social sciences feature a pervasive but problematic meta-assumption that is characterized by an ‘all-seeing eye’” (Felin et al., p. 1).

We take the essence of this idea to be the assumption that there is an objective, absolute “view from nowhere” (Nagel, 1989) of relevant aspects of the physical or social world, and that the theorist must adopt this Archimedean standpoint to provide a commentary and critique on observed thought and behavior. In particular, Felin et al. suggest, advocates and detractors of rational models of human decision making and perception implicitly assume that “rationally correct” performance can be judged from an objective standpoint of this kind.

Theorists differ concerning whether human rationality is a glass half full or half empty. Rational-choice theorists in the social sciences and proponents of Bayesian models of perception and cognition focus on cases in which human thought and behavior matches up well with rational standards. By contrast, those who focus primarily on the limits of rationality, whether in the study of human reasoning, judgment and decision making, or behavioral economics, use this presumed objective standpoint as an external standard against which our behavior can be measured and found wanting. Felin et al. argue that an objective “all-seeing eye” may be a mirage—and that, in consequence, debates on rationality and the social sciences may need to be substantially rethought.

In this commentary, we argue that rational explanation need not, and typically does not, rely on a hidden assumption of the existence of an “all-seeing eye.” Our argument has four steps. First, in the Functional Versus Equilibrium Explanation section, we distinguish two very different styles of explanation. Second, in Rationality in Functional and Equilibrium Explanation, we describe how rationality can play a role in these two very different styles of explanation. Third, in The All-Seeing-Eye in Rational Explanation, we argue that Felin et al.’s critical arguments apply only to rational functional explanation (which is prevalent in vision research) but not to the rational equilibrium explanation (which is prevalent in the field of judgment and decision making). Fourth, in The Scope and Limits of Rational Functional Explanation, we argue that although Felin et al. are right to point out potentially strong assumptions in the application of rational functional explanation (e.g., as used in rational models of perception), the scope for such explanation is nonetheless very large. Indeed, just as with conventional functional explanation in biology, the rational functional explanation of cognitive processes has considerable explanatory power.

***Functional versus equilibrium explanationtpb***

Explanation in many aspects of the biological sciences is paradigmatically functional (Tinbergen, 1963). The heart has the function of pumping blood; arteries and veins have the function of diffusing oxygen and nutrients efficiently through the body; the lungs have the function of efficiently exchanging oxygen and carbon dioxide. Functional explanation extends to the microscopic scale, as well: The cell wall has the function of maintaining a stable chemical environment; the myelin sheath around nerve axons has the function of preventing the dissipation of the electrical pulse created by depolarization; synapses have the role of linking together of nerve cells, perhaps in a way that can be adjusted through experience; and so on. Each of these functional explanations is partial. As with explanation in most domains, functional explanations can be made increasingly nuanced and sophisticated—taking account, for example, of multiple competing functions, or of constraints from ontogeny and phylogeny, as well as physics and chemistry. Biology is functional through and through: Each element of a biological system is understood, in part at least, as contributing to the successful operation of a larger system of which it is a component.

Explanation in microeconomics is, by contrast, paradigmatically focused on equilibrium, not function. Prices are set so that supply balances demand. Consumers are presumed to distribute their spending so that the marginal utility of an extra dollar is in precise balance across a variety of goods; similarly, companies are presumed to distribute their resources to land, machinery, labor, and so on, so that the marginal impact of each extra dollar spent on these various factors of production is precisely in balance (e.g., Kreps, 1990). In the same way, in finance theory, each stock and bond price is presumed to reflect the same balance between risk and return (Sharpe, 1964). In each of these cases, the focus is bringing the parts of a single system into equilibrium, whether it be trade between buyers and sellers, consumer spending across different categories, company investment across factors of production, or prices across the stock market.

Functional and equilibrium explanation are profoundly different. Functional explanation sees the system under study as having a role in a larger system, and explains the properties of the system under study by reference to the degree to which it successfully performs this role. For example, the heart is seen as playing the crucial role of pumping blood through the body; the circulatory system, more broadly, is itself seen as having a role in the processes of respiration and digestion; these in turn are seen as critical to supporting the physiological processes, neural function, and the behavior of the organism. From the perspective of natural selection, the chain of functional explanation may, arguably, be grounded in an “overarching” goal of maximizing inclusive fitness—that is, the function of maximizing the rate of reproduction of individuals, or more accurately the genes from which they are constructed (Dawkins, 1976; Hamilton, 1964). Crucially, explaining a system by its function requires holding that system to a standard that can be defined from outside the system itself.

Equilibrium explanation, by contrast, focuses not on the degree to which a system carries out some externally defined function, but rather on bringing into balance the components within the system itself. The focus of interest is not external “success” but internal balance or coherence. Often, where the system is assumed to be near equilibrium, the question of interest is the extent to which equilibrium can help predict the direction in which the system will change over time. For example, equilibrium explanation in economics links together prices of crude oil, petroleum, diesel, and indirectly of other sources of energy, to the valuations of oil companies, motor manufacturers, and so on. Thus, if there is a shock to the supply or likely future availability of crude oil and a consequent spike in the crude oil price, the price system is assumed to transmit this shock throughout the economy to set prices in a new equilibrium.

***Rationality in functional and equilibrium explanation***

Crucially for Felin et al.’s discussion, *rational* explanations can have both functional and equilibrium forms. Rational *functional* explanation arises where the function of interest is not a physical or chemical process, as in digestion or respiration, but an information-processing function, whether this is making arithmetic calculations, understanding speech, interpreting sensory input, or controlling the motor system. Such explanation involves the theorist taking an external standpoint and creating an “ideal” rational model that addresses the problem that the theorist takes the agent to be facing: its goal, environment, and perhaps its cognitive limitations (Anderson, 1990, 1991). The hope is that the predictions of the ideal model will fit, to some degree, with the observed behavior. The external standpoint is exemplified in behavioral ecology (Krebs & Davies, 1978); Marr’s (1982) computational level of explanation; rational models of memory, categorization, and reasoning (Anderson, 1990, 1991; Oaksford & Chater, 1994, 1998; Tenenbaum & Griffiths, 2001); “ideal observer” models of perception (e.g., Geisler, 2011; Yuille & Kersten, 2006); Bayesian theories of perception and motor control (e.g., Körding, & Wolpert,^ 2006^; Yuille & Kersten, 2006); and ideal models in language processing or acquisition (e.g., Chater & Manning, 2006; Pinker, 1979; Vitányi & Chater, 2017). In rational functional explanation, a well-defined information-processing task is to be performed that can be characterized from outside the system; the rational functional model provides an optimal solution to performing that task.

Note that, as with functional explanation more broadly, rational functional explanation does not require that the organism as a whole be fully optimal or close to optimal with respect to any externally defined function. Indeed, functional explanations typically involve local rather than global optimization. Thus, the optics of the eye seem locally fairly optimal, in the sense that any small change to the structure of the lens, the cornea, or the retina is likely to lead to poorer, rather than better, optics; but there is no implication that the vertebrate eye, considered as a whole, is somehow the “ideal” optical system. Similarly, the rational functional explanation of animal foraging typically assumes that a local change to foraging patterns (e.g., switching “patches” more or less frequently) should not be advantageous; but there is no implication that the agent has achieved, in some sense, the ideal possible foraging method.

The all-seeing eye of Felin et al. seems, to a degree, to be implicated in functional rational explanations, and as we discuss further below, Felin et al. are right to point out that there are many aspects of perception and cognition for which no meaningful “objective” goal is being optimized or “true” description of the world is to be extracted.

Felin et al.’s concerns about the “all-seeing eye” do not, however, apply to rational *equilibrium* explanation. In equilibrium explanation, the theorist does not attempt to stand outside the cognitive system under study. Rather than taking the nature of the problem faced by the agent as the starting point for analysis, equilibrium explanations take the beliefs, preferences, and actions of the agent as the theoretical starting point, and use rational principles to try to weave these together into a coherent whole. The analogue of microeconomic explanation as an equilibrium explanation of markets is rational choice theory as an equilibrium explanation of individual behavior (e.g., Becker, 1976). Rational choice is the standard model of individual behavior in economics—and it is this equilibrium style of explanation that is under scrutiny in the literatures of judgment and decision making (e.g., Kahneman & Tversky, 1984) and behavioral economics (e.g., Camerer, Loewenstein, & Rabin, 2011).

Consider, for example, the notion of utility in modern economic theory. Rather than seeing utility as corresponding to some externally measurable good or objective (as in earlier economic ideas; Cooter & Rappoport, 1984), the modern notion of utility in economics serves simply to organize the preferences of an agent into a compact and coherent form. For example, a preference for coffee over tea, tea over milk, and coffee over milk can be captured by assigning utilities of, say, 3, 2, and 1 to coffee, tea, and milk, respectively, and proposing that the agent prefers drinks with a higher utility. Some rather mild constraints on the structure of preferences (e.g., most notably, completeness and transitivity) will ensure that such a utility function can be defined.

Note, crucially, that the very idea of utility is in no way concerned with performance according to some externally defined function; instead, it merely imposes internal coherence on an agent’s own preferences. There are good reasons to have preferences that have such coherence, and hence that allow a utility scale to be defined. For example, if an agent’s preferences are *in*transitive (preferring, say, coffee to tea, tea to milk, and milk to coffee), and hence cannot be represented by utilities, then that agent can be turned into a so-called “money pump.” Suppose that the agent starts with coffee. A devious counterparty can then offer to swap the coffee for milk, for a small fee; and then to swap milk for tea for a small fee, and finally to swap the tea back to coffee for a small fee. Now the agent is back where he or she started, but has paid fees to no purpose. Unless the agent changes one of the offending preferences, this can be repeated indefinitely until the agent’s resources are exhausted (although see Cubitt & Sugden, 2001). So, even in the absence of any externally defined task, it seems that intransitive preferences must violate some form of rationality.

If we broaden our agent’s domain to being able to trade uncertain options (e.g., tea if the teaspoon falls face down, or coffee otherwise), then on some similarly mild conditions (completeness, transitivity, and some more technical assumptions about independence and continuity), the agent can be assigned *utilities* (about tea, coffee, and so on) *and probabilities* (e.g., degrees of belief in the teaspoon falling face down), such that one uncertain option is preferred to another just when it has a higher expected utility, according to those utilities and probabilities. Then it turns out that if our agent makes any violation of probability theory, whether large or small (e.g., perhaps the agent commits the conjunction fallacy; Tversky & Kahneman, 1983), then the agent can also be money-pumped: Specifically, the agent can be offered a set of gambles, each of which the agent believes is fair but that collectively guarantee that the agent will lose money (this is known as a “Dutch book”). Again, rationality is a matter of coherence within the agent: here, linking the agent’s degrees of belief, utilities, and actions (i.e., choosing one option or another). Note this equilibrium notion of rationality is what is at issue in the fields of judgment and decision making and behavioral economics, where the standard against which human behavior is judged is drawn from abstract theories of coherence: probability theory, decision theory, game theory, and logic (e.g., Chater & Oaksford, 2012).

***The all-seeing eye in rational explanation***

Felin et al. put forward an argument concerning hidden assumptions underlying rational explanation in the context of vision, and then extrapolate to a discussion of rationality in economic decision making. We suggest, however, that the style of explanation and the role of rationality are very different in each case, and, crucially, that this blocks the extrapolation from one domain to the other. Felin et al.’s concern is, in brief, that it is mistaken to suppose that the function of perception is to “reconstruct” some objectively described “external world” from sensory data. They rightly point out that many aspects of perceptual experience (e.g., color, lightness, and, we would argue, most everyday common-sense categories for which there are verbal labels) no more correspond to the nature of the external world than, to borrow Hoffman’s (2009) powerful analogy, the colors, shapes, and layouts of items on a computer desktop correspond to objective internal states of its silicon chip.

Yet no such characterization of the external world is required in order to make sense of rational equilibrium explanation, which depends purely on the coherence of the internal elements of the system. Note, for example, that the rational principles of finance theory impose coherence constraints on prices, but without requiring any particular relationship between prices and the external world. For example, it could be that the entire market is vastly overpriced if, for example, no market participants are aware of an imminent exogenous “shock”—such as a cataclysmic meteor strike. Of course, were astronomers to declare such a strike to be likely, stock market panic would ensue—the disequilibrium between stock valuations based on putative long-term revenue streams and the apparently short-term time horizon of civilization would lead to a market collapse. But, tellingly, it is the beliefs of astronomers and investors that matter, not the actual state of the external world (so that the panic will be just as great, even if the meteor strike is actually a false alarm).

Rational explanation in judgment and decision making works in the same way as in finance theory or microeconomics. The conjunction fallacy, for example, exposes an incoherence between degrees of belief (i.e., that a person believes A & B to be strictly more likely than A alone). But any external reality to which propositions A and B refer is irrelevant. The essence of mathematical theories of rationality, such as probability theory, decision theory, game theory, and mathematical logic, is that they describe an incoherence between beliefs, preferences, or actions that is purely structural in nature. Matters of internal coherence (or incoherence) are entirely independent of external reality. Hence, Felin et al.’s concerns about the all-seeing eye do not arise.

***The scope and limits of functional explanation***

Functional explanation, whether involving rationality or not, aims to understand the function of a system within some large system, and hence the theorist needs to be able to characterize the nature of that larger system. Here, Felin et al.’s concerns have potential bite. Can the larger external system always be characterized? And can it be characterized independently from the system under study?

We agree that these are deep issues. But they are not specific to *rational* functional explanation (e.g., ideal observer models, Bayesian models of perception, Marr’s computational level of explanation, and so on), but arise for functional explanation of any form. Thus, viewing the heart as functioning as a pump requires outlining other elements of the circulatory system. And viewing a biological structure as consisting of arteries or veins (rather than a mere network of flexible tubes) may make no sense, except in light of their connections to the heart. Similarly, the courtship behavior of a bird has the function of increasing the probability of mating, yet that behavior operates not via shaping some objective external reality, but via the interpretation of that behavior by a potential mate. More broadly, the behavior of a species may be well-adapted to functioning successfully in its niche, but that niche may not necessarily have an existence wholly independent of the species itself.

In practice, despite these deep issues, functional explanation in biology appears to be hugely valuable throughout the biological sciences: Seeing the heart as a pump, or courtship behavior as driven by mate-finding, seems extremely productive, even essential. And viewing stereopsis or structure-from-motion as mechanisms that aim to recover the 3-D layout of the world, or viewing memory as adapted to prioritizing information that is likely to be useful in the current situation, seem no more (or less) problematic than conventional functional explanations concerning, for example, the shape of the lens or the transparency of the cornea.

*Conclusion*

Felin et al. raise important concerns with the application of rational explanation in the cognitive and social sciences. We argue that rationality plays a part in two very different styles of explanation: functional explanation (where Felin et al.’s concerns potentially apply) and equilibrium explanation (where they do not). Even where Felin et al.’s worries do apply, and theorists need to provide an external characterization of the “environment” or “task” for the perceptual or cognitive process under study, functional explanation is often feasible and valuable—indeed, we believe it raises no special difficulties over and above the wider challenge of making sense of functional explanation in the biological and social sciences.

**Author note** N.C. was supported by ERC Grant 295917-RATIONALITY, the ESRC Network for Integrated Behavioural Science [Grant No. ES/K002201/1], the Leverhulme Trust [Grant No. RP2012-V-022], and Research Councils UK Grant EP/K039830/1.

**Who are you going to believe? A comment**

David Funder

*Who are you going to believe: Me, or your lying eyes?*

*—Anonymous*

If I may use an auditory metaphor on top of the visual one used so effectively by Felin et al. (2017), the term “multistable rationality” sounds disturbingly similar, in my ears, to the term “alternative facts” recently popularized in US political discourse. And just as taking the idea of alternative facts seriously will lead to ruin, so too, I fear, will the idea of multistable rationality.

I agree with the article in many respects, particularly its calling into question the unrealistic and even wrong standards of rationality that are often used as the basis for regarding human judgment as deeply flawed. I also think the article is profoundly correct to note how human judgment is typically evaluated not only against questionable criteria, but against a standard of perfection. It is telling that, in research from the errors-and-biases tradition, *any* deviation from the normative model employed is held up as evidence of flawed judgment. Even if the criteria for correct or rational judgment were always justified, which they are not, the news from the vast literature on judgmental error would still boil down to “people are not perfect.” And this is news? More useful, perhaps, might be to study how people get things right, when they do (Funder, 1995, 1999; see Felin et al., note 18).

Still, the target article goes too far in its criticism of the visual metaphor as a way to see reality. I did not find the most vivid example in the article to be compelling. When someone in a gorilla suit walks by a person counting basketball passes and is not noticed, that is an amusing demonstration of focused attention. As the authors note, it does not imply that people are blind, or stupid. But neither does it show that reality in this situation cannot be visually identified. Someone looking at the gorilla might miscount the passes; someone counting the passes might overlook the gorilla. But a detached observer could, in principle, look at both, or at least could look at one and then the other, and get a more full view of reality. We don’t always, or perhaps ever, need to know about everything that is happening around us, fortunately. But it can be done; indeed, the paragraph in the article that points out the alternative views of the gorilla-blindness situation could not have been written if this were not so.

I will grant that we see only a small part of the electromagnetic spectrum, and that we evolved as a species to survive and reproduce, not to accurately represent reality. But these goals are surely compatible, and perhaps even mutually necessary; to some extent survival must require accurate apprehension of reality. Deep philosophy and quantum theory aside, I would suggest that the portion of the environment susceptible to human observation is, for human purposes, a sufficient definition of reality.

Indeed, to conclude otherwise is to undermine the foundations of science itself, an enterprise that outside the academy is making disturbing progress these days. As many observers have noted, science is a sort of game that is designed to get us ever closer to the truth, while at the same time recognizing that we shall never quite get there. But if truth doesn’t exist, then getting closer to it has no meaning.

Gordon Allport (1958) once noted how some psychologists wished to regard personality traits as hypothetical constructs that don’t exist in any real sense, but are merely thoughts in the minds of observers. He acknowledged that this view has some merit, because traits cannot be directly seen (that visual metaphor again), but only implied from indirect indicators such as self-reports and behavioral observations. But then, Allport suggested an analogy with astronomy. Those little lights in the night sky, images in our lenses, and pulses detected by radio telescopes are at best extremely indirect indicators of the stars and galaxies that are presumably really out there. But if we took away that presumption of reality, and decided that stars and galaxies are no more than hypothetical constructs in the minds of astronomers, then astronomy would become the study of how astronomers think, rather than the study of the contents of the universe. Indeed, it might be interesting to study how astronomers think. But it would be sad to stop trying to learn about what’s really out there. By the same token, Allport pleaded for a psychology that tried to learn what it could about *personality*, not just people’s perceptions of it.

I feel the same way about multistable rationality. I’m happy to argue about alternative definitions of reality, or of rationality, but I’d like the goal of the argument to be to decide which one is right, even if the goal is never achieved. In my own work, something I call the “situation construal model” identifies the individual construal of reality as a crucial determinant of his or her behavior (Funder, 2016). This construal is a joint function of the individual’s personality and cognitive traits (individual differences), and reality itself. The model puts alternative construals at the center of the analysis, but gives reality a critical role too.

What is reality? This is the oldest, deepest and most unsolved question in philosophy. A common response to the question is to give up. Cultural anthropologists often eschew comparing cultures with each other because they acknowledge lacking the god’s-eye view that would allow them to unerringly do so. Deconstructionist literary critics maintain that texts have no inherent meanings, just an infinite number of equally valid constructions thereof. Shall the psychological study of human judgment fall into the same trap? For I believe it is a trap. Reality is hard to know; we can never be sure we know the truth. But to give up the attempt to seek truth is not a solution; it is a surrender, one that strands us in a world where alternative facts rule.

**Visual illusions and ecological rationality**

Gerd Gigerenzer

According to Helmholtz, visual illusions reveal the ingenuity of the visual system, namely its ability to make intelligent unconscious inferences from limited or ambiguous information. These illusions help unravel the remarkable feats our brain can accomplish. In striking contrast, behavioral economists and some psychologists have presented visual illusions to support their claim that the mind systematically lacks rationality. If the visual system continues to make consistent errors after millions of years of evolution, what can be expected from human judgment or business decisions? Tversky and Kahneman (1974) argued that people make severe errors of judgment, later named *cognitive illusions*, in analogy to visual illusions. Today, rare is the book in behavioral economics that does not refer to visual illusions in order to describe cognitive illusions, mistaking the ingenuity of the visual system for irrationality. For instance, in *Phishing for Phools*, Akerlof and Shiller (2015) tell us that “psychological phools” come in two types: “In one case, emotions override the dictates of his common sense. In the other case, cognitive biases, which are like optical illusions, lead him to misinterpret reality” (p. xi). Because visual illusions are persistent, so the argument goes, people’s cognitive illusions are equally stubborn, leaving little hope of any sustainable corrective. Out of this perspective, visual illusions became the justification for governmental paternalism, known as *nudging* (Thaler & Sunstein, 2008).

How could vision be suddenly mistaken as the prototype of stubborn irrationality? Felin, Koenderink, and Krueger (2017) point to a specific assumption in this research, which they call the *all-seeing eye*. The assumption is that the researcher is gifted with omniscience and always able to see the correct state of the world or the correct answer to the text problem studied. In the Müller-Lyer illusion, the correct state is assumed to be the physical lengths of the lines: “The presence of an error of judgment is demonstrated by comparing people’s response either with an established fact (e.g., that the two lines are equal in length) or with an accepted rule of arithmetic, logic, or statistics” (Kahneman & Tversky, 1982, p. 123). Felin et al. criticize this narrow understanding of what the “fact” or benchmark for a perceptual judgment is and, consequently, how such an assumption misses the very function of perception: to go beyond the information given on the retina (or in a drawing), that is, to infer the third dimension from a two-dimensional picture. To equate measured dimensions with statistical rules also claims a second assumption, namely researchers’ omniscience regarding the correct answer to problems that involve probability or statistics (the so-called “accepted” rule). I agree with Felin et al.’s critique of this fact-minus-judgment analysis, which is a step backward from both Helmholtz and present-day research on perception (e.g., Gigerenzer, 1991, 2005). However, they appear to think that the assumption of omniscience also underlies the study of ecological rationality (Todd, Gigerenzer, & the ABC Research Group, 2012) when writing that ecological rationality assumes “that perception, over time, is in fact veridical rather than biased: organisms . . . learn its true, objective nature” (p. 2). I am grateful for their making this misunderstanding explicit because it provides an opportunity to clarify what ecological rationality is: It means functionality, not veridicality. To address this misunderstanding, I will briefly discuss one concrete visual illusion.

***Seeing is inference***

Consider Fig. 1. On the left side are dots that appear concave, that is, recede into the surface like cavities. On the right side are also dots, but these appear convex, that is, pop out from the surface like eggs. Now turn the page upside down. The cavities will turn into eggs, and the eggs into cavities. This striking phenomenon is a key to understanding the workings of the human brain (Kleffner & Ramachandran, 1992).

**Fig. 1:**
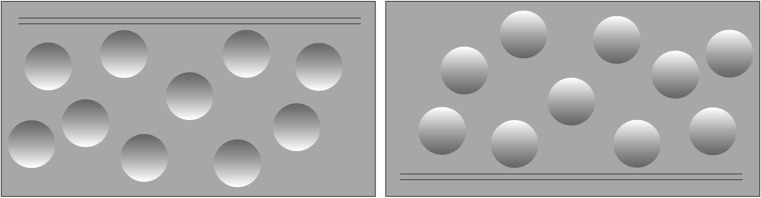
Visual Illusions illustrate unconscious inferences on the basis of assumptions about the environment. The dots on the left side appear to be curved inward (concave), those on the right side curved outward (convex). If you turn the page around, then the concave dots will pop out and the convex dots will pop in (based on Kleffner & Ramachandran, 1992)

The brain cannot know the outside world with certainty; it can only make intelligent inferences. In the present case, it faces an impossible task: to construct a three-dimensional object from its two-dimensional retinal image. How can this be achieved? To infer what is outside, the brain uses a chain of intelligent inferences, based on three assumptions about the world:

Ecological Axiom 1: *The world is 3-D.*

Ecological Axiom 2: *Light comes from above.*

Ecological Axiom 3: *There is only one source of light.*

I call these assumptions ecological axioms because, unlike the logical axioms of decision theory, these statements have content and are based on the ecological structure of the world. The three ecological axioms are characteristic for human and mammalian history. We can move in space south–north, west–east, and up–down, that is, in three dimensions (3-D), and for most of mammalian history, light came from a single source above, the sun or moon. Even today, we sit in rooms in which the light predominantly comes from above, and we may get confused by a light coming at us horizontally, such as by the headlights of another vehicle while driving, or by multiple lights coming from different angles.

These three axioms help us to infer the third dimension. Yet this inference is ecological rather than purely logical, using a heuristic, that is, a simple rule that exploits environmental structures. When light falls on a three-dimensional object, its rays cast shadows. Our brains use this relationship to infer the third dimension from the position of the shading:

Shade heuristic: *If the shade is in the upper part of the dots, then they recede into the surface; if the shade is in the lower part, the dots projects out from the surface.*

Now consider Fig. 1 again. On the left side, in which the upper part of the spheres is darker, we see the spheres as cavities. On the right side, in which the lower part is darker, we see them popping out toward us. The position of the shading is sufficient for making the inference. Now let us have a closer look at the two concepts in the if-clause of the heuristic, *shade* and *upper part*. Our brain cannot know for certain that a dark area is a shadow and thus has to make another intelligent inference. Again it uses cues. The decisive cue appears to be the fuzziness of the border between the dark and light parts. Shadows are fuzzy. One can experimentally test this explanation by replacing the fuzzy contour with a clean-cut black-and-white contour. When the fuzziness is removed, our brain no longer interprets the black color as shade, meaning that the if-clause of the shade heuristic is no longer valid (Ramachandran & Rogers-Ramachandran, 2008). In the same way one can investigate how the brain infers what “upper part” means. Is “up” relative to the ground (gravity) or relative to the position of one’s head? Experiments show that the brain infers “above” from the position of the head, that is, using its vestibular system rather than gravity. Signals from the body’s center of balance—the vestibular system—guided by the positions of little stones in the ears called otoliths, travel to the visual centers to correct the mental picture of the world (so that the world continues to look upright).

To summarize, seeing a dot as concave or convex appears to be based on three ecological axioms and the shade heuristic, which in turn is based on a cascade of inferences that estimate the concepts in the if-clause of the heuristic. These inferences invoke the vestibular system (to infer where “above” is) and contour detection systems (to infer that there is a shadow). The example also illustrates how heuristics feed on evolved core capacities of our brain.

***Ecological rationality***

So far, I have introduced three concepts for understanding of perceptual inferences: *uncertainty*, *ecological axioms*, and *heuristic inference*. The final concept is:

Ecological rationality: *A heuristic is ecologically rational to the degree it is adapted to the environment, that is, to the conditions specified in the ecological axioms.*

The term *environment*, as defined by the ecological axioms, does not relate to a world independent of humans, as Felin et al. appear to assume, but to the world as experienced by humans, as in von Uexküll’s (1957) *Umwelt*. For instance, the axiom that the world is 3-D is not a statement about the true structure of space, but a statement about the fact that humans experience a three-dimensional world in the sense that we can move right–left, forwards–backwards, and up–down. In Fig. 1, the 3-D axiom is violated because this world has been replaced by a two-dimensional drawing. In this specific setting, using the shade heuristic is not ecologically rational, but in a three-dimensional world, it is. The resulting discrepancy is often called a *visual illusion*. Yet it is the result of an intelligent inference under *uncertainty* that should not be confused with irrationality.

***Uncertainty is inescapable***

Uncertainty about the world in which we live is not restricted to the third dimension in space, as in the example above. It is universal because our sensory systems provide a limited basis to construct the world, in several respects: (1) *Limited senses.* For instance, humans cannot sense electrical and magnetic fields or radioactivity, nor can they detect water pressure. And we may lack all those senses we are unaware of, such as that indicated by animals’ “knowing” an earthquake is coming before we can notice anything. (2) *Limited range within a sense.* Our senses have absolute upper and lower thresholds, beyond which we do not notice anything. (3) *Limited discriminability within a range.* Our senses have differential thresholds, which are defined as the just noticeable difference. (4) *Limited samples of experience*, due to our finite attention and lifespan. Small samples can be exploited by simple heuristics, which is another reason why the brain often works with heuristics rather than fine-tuned complex algorithms (Gigerenzer, 2016). These heuristics are functional, not veridical, and no omniscient researcher is needed to study the ingenuity of visual intelligence.

All these are characteristics that define the “twilight of uncertainty,” which contrasts with the assumption of an all-seeing eye. Felin et al. have reminded psychologists that there are two kinds of research questions one can pose. The first is about uncertainty: How does the brain construct its world in order to be able to function, replicate, and survive? The second is about certainty: Do people’s judgments deviate from what I, the researcher, believe to be the correct, and only correct, answer? Asking the first question has brought deep insights into the workings of the mind–environment system. Asking the second question has resulted in a list of 175 cognitive biases that can be found on Wikipedia, with little understanding of their precise mechanism and function. To find the right answer to the wrong question is known as a Type III error. There are still too many of these in our journals.

**Biological relativism and directed perception**

Denis Noble

Felin, Koenderink, and Krueger’s (2017) article is a highly welcome counter to a persistent, but incorrect, twentieth-century development in the science of perception. This is the idea that to analyze perception we need to understand simply that there is a mismatch between how science objectively describes the world and how humans (and other organisms) perceive it. We then explain visual and other illusions by comparing them with objective measurements such as wavelengths of light or other radiation.

By contrast, Felin et al. challenge the idea that any fully “objective measurement of color or luminance” is possible since “it is not as easy (if not impossible) to disentangle illusion, perception, and reality”. They then show how the metaphors derived from the physiology of perception have influenced our understanding of rationality, and rational agency in particular, as used in economics and other areas of social science. Their position resembles in part von Uexküll’s and Tinbergen’s ideas that each organism has its own unique way of viewing the world, for identifying objects and their utilities, and associated perceptions and behaviors. As Felin et al. noted, “perception therefore depends more on the nature of the organism than on the nature of the environment”. And, the “fundamental issue is the directedness of perception due to a priori factors associated with the organism itself”. I would particularly like to emphasize their point about the “directedness” of perception, to anticipate my conclusion.

One of the reasons I support Felin et al.’s conclusions is because their argument has a lot in common with a major argument currently occurring in biological science. This is on the nature of biological causation. The strong form is represented by the view that there is an objective description of what organisms perceive, and thus organisms are essentially passive agents in the world. Causation runs in one direction: upward from molecules to man. This view can be traced back at least to Descartes in 1664:

If one had a proper knowledge of all the parts of the semen of some species of animal in particular, for example of man, one might be able to deduce the whole form and configuration of each of its members from this alone, by means of entirely mathematical and certain arguments, the complete figure and the conformation of its members. (*De la formation du fœtus*, para LXVI, p. 146)

We can trace this kind of argument all the way from Descartes’ text through Laplace (1840/2003), to Schrödinger (1944), to the central dogma of molecular biology (Crick 1958)—a historical sequence that I have analyzed and countered in a recent book (Noble, 2016, chap. 6).

Modern neurophysiology has revealed the many respects in which causation runs centrifugally *from* the central nervous system to modulate input from the sense organs. But there is still a presumption that, behind all this modulation and interpretation, the result is still a *representation* of reality. In principle, it could be made to conform to the assumed objective reality of physical measurements, once we allow for the mechanisms that create the illusions. The one (perception) is simply a transform of the other (reality). Reality can then in turn be seen as a transform of what we perceive. We just need to know the transform function and we can then know the reality.

The more fundamental question is whether an objective reality is waiting there to be perceived. This is no longer as obvious as was once assumed. At the least, modern physics warns us that “reality is not what it seems,” to quote the title of Carlo Rovelli’s (2016) magnificent book.

So, how did we come to think otherwise? In the 19th century, both physics and biology assumed there was a clear objective reality; indeed it was thought to be so obvious as not to need stating. At the beginning of the 20th century physics peeled away from that view. Relativity and quantum mechanics taught us that we were not at all sure what reality we might be talking about. In the 20th century, however, biology largely ignored these developments in physics. Quantum mechanics was thought to concern only the microphysical level, whereas relativity concerns only the cosmic scale.

I take a more radical view, which is that it was unfortunate that the revolution in physics passed by with little or no impact on biological science. My first reason is that the revolution that led to quantum mechanics destroyed the assumption that the bottom level in scientific explanations of phenomena is a rock-solid atomism. Molecules, such as genes and proteins, may not be the ultimate bottom level, but they could be assumed to rest on lower levels that were seen as determinate, or hard, an assumption that was needed for the reductionist agenda to be successful. We now know that this is simply not the case. As we descend the levels below atoms and molecules, we encounter stochasticity, not certainty, in the behavior of the universe.

My second reason is that biology needs to follow physics in respecting the general principle of relativity. Here I need to explain what the “general principle of relativity” is. Relativity is usually identified today with the two theories of Einstein: the special and general theories of relativity. These are indeed examples of the general principle of relativity, which is the strategy to distance ourselves from any metaphysical standpoint for which there is insufficient justification. Thus, the special theory of relativity can be seen as distancing us from the assumption of a privileged spatiotemporal frame of reference, from which it becomes clear that there is no absolute measure of movement. The general theory of relativity can be seen as distancing ourselves from the assumption that space–time and gravity are distinct. These paradigm shifts can also be seen to rest on previous applications of the general *principle* of relativity, which first moved us away from regarding the earth as the center of the universe, then from the idea that the sun might be, and finally to the idea that there is no center—and no edge either.

The unjustified metaphysical idea in biology is that there is a privileged level of causation, which was assumed almost without question to be the molecular level. This metaphysical position, for that is what it is, led in turn to the modern strongly gene-centric view of biology. In this view, agency in biology ultimately derives from genes, which are the objects of natural selection. In its more extreme forms this view actually removes agency from organisms. They become Cartesian automata. This is the view taken by many gene-centric biologists as a natural interpretation of, for example, the experiments of Libet, Gleason, Wright, and Pearl (1983) on the neural basis of decision (Noble, 2016, pp. 247–267).

It was, in part, the absurdity of this view of agency in organisms that led me to formulate the principle of biological relativity. This states that, a priori, there is no privileged level of causation in biology. Like other theories of relativity, the principle of biological relativity is a deeply mathematical concept. Another way to state the principle is that the equations of any model we may construct to describe biological systems depend on the initial and boundary conditions for any solution of the equations to be possible. The initial conditions are a function of the history of the organism, whereas the boundary conditions are a function of the environment and its interaction with the organism.

This principle leads to a view of agency in organisms that is, I believe, strongly compatible with the view propounded by Felin et al. Organisms are then viewed as agents creating their environment, and as agents in the directedness of their own evolution.

That is why I strongly agree with Felin et al. that the “fundamental issue is the *directedness* of perception due to a priori factors associated with the organism itself”.

**Ecological rationality needs no all-seeing eye**

Samuel Nordli, Peter M. Todd, Gerd Gigerenzer

Rationality has been envisioned in multiple ways. Traditional unbounded views of rationality derive optimal decisions by assuming unlimited knowledge and unlimited cognitive power to process that knowledge; constrained optimization views of rationality add some cognitive limitations but still assume a psychologically implausible process that aims for the best possible decisions under those constraints. Visions of bounded rationality focus on plausible heuristics that people and other animals may use. In the heuristics-and-biases approach (Kahneman, Slovic, & Tversky, 1982), such heuristic decision making is seen as often leading to biases and deviations from optimal behavior; in contrast, the ecological rationality framework analyzes which structures of environments enable heuristics to achieve adaptive goals. Felin, Koenderink, and Krueger (2017) make the interesting observation that most of these visions of rationality imply a capacity for unlimited perception—the all-seeing eye—which permits the identification of optimal decisions with its single veridical assessment of the entire state of the world. However, contrary to what Felin et al. at times suggest, this applies to neither the vision of ecological rationality nor Herbert Simon’s vision of bounded rationality. In this commentary we address how Felin et al. appear to conflate the bounded rationality championed by Simon with that of Kahneman, Tversky, and others; we go on to show how this leads Felin et al. to mischaracterize the Simon-inspired framework of ecological rationality. We then explain how the vision of ecological rationality provides a natural cognitive complement to the ideas of limited, specific perception that Felin et al. champion; finally, we highlight how limited perception can be a cognitive advantage and why the empirical investigation of general cognitive capacities is not as fruitless or futile as Felin et al. make it out to be.

***Simon’s bounded rationality is not Kahneman’s bounded rationality; ecological rationality is not optimization***

It is important to distinguish two types of research questions when studying rationality and decision making. The first asks how people make decisions in situations of *uncertainty—*that is, when the future is uncertain and one cannot know the best answer ahead (see Todd, Gigerenzer, & the ABC Research Group, 2012); under uncertainty, by definition, omniscience is a fiction and optimal strategies cannot be calculated. The second type assumes a situation of *calculable risk*, removing all uncertainty (Knight, 1921) and allowing for the convenient mathematics of optimization. In psychology, optimization models have been used to argue in favor of human rationality, as in Bayesian models of cognition, but also to argue for our lack of rationality, as in the heuristics-and-biases program. Felin et al. charge that optimization models unjustifiably assume a veridical assessment of the entire state of a given situation, as that assumption requires the impossible all-seeing eye. We agree that this is a major and problematic assumption in the heuristics-and-biases program of Kahneman, Tversky, and others. For virtually every problem posed to subjects, these researchers presume to know the correct answer a priori, although this self-declared omniscience has not stood up to critical tests (e.g., Gigerenzer, Fiedler, & Olsson, 2012; Hertwig & Gigerenzer, 1999). When responses deviate from these omniscient projections, such “errors” are typically attributed to cognitive illusions. Felin et al.’s critique of many psychologists’ overconfidence in their normative convictions is timely: researchers must distinguish between situations of risk, in which the best answer can be calculated, and those of uncertainty, wherein optimization is impossible—except in hindsight.

Yet Felin et al. appear to have been misled by a wider literature that misrepresents Simon as a genuine precursor of the heuristics-and-biases approach. The latter approach accepts the classical norms of economic theory as rational, claiming irrationality when human judgments deviate from these norms; in contrast, Simon argued that the study of bounded rationality should deal with situations of uncertainty in which “the conditions for rationality postulated by the model of neoclassical economics are not met” (Simon, 1989, p. 377). Simon advocated acknowledging the limits of optimization; for him, the use of heuristics is not a deviation from what is optimal, but rather an example of how people satisfice when they cannot optimize. Felin et al. are mistaken when they equate Simon’s call for studying behavior under uncertainty with the assumed omniscience in the heuristics-and-biases program—the all-seeing eye as depicted by Felin et al. does not feature in the program of bounded rationality promoted by Simon. As a direct consequence of this confusion, the guilt of presumed omniscience is similarly misattributed to those operating within the framework of ecological rationality (Todd, Gigerenzer, & the ABC Research Group, 2012). Directly building on Simon’s program of bounded rationality, this framework studies both (1) the heuristics people use when optimal answers are indeterminable and (2) the ecological conditions in which a given heuristic can be expected to outperform competing strategies (even those using relatively more information).

The overall perspective of ecological rationality is that adaptive decision-making can emerge from the fit between the structures of appropriate information-processing mechanisms in the mind and the structures of information in the world. These mental mechanisms are often simple heuristics that exploit the available structure of environments, using relatively little information to reach good-enough (not optimal) solutions to the challenges facing the organism (Todd & Gigerenzer, 2007). One of the implications of this foundation is that perception need not be veridical—it only needs to be effective for the adaptive problems at hand, just like the decision-making process overall: “for cognition to be successful, there is no need for a perfect mental image of the environment—just as a useful mental model is not a veridical copy of the world, but provides key abstractions while ignoring the rest” (Todd & Gigerenzer, 2012, p. 15). Perceptual illusions then can show which environmental structures are important and useful to (and hence assumed by) the particular cognitive system. For instance, in the concave/convex dots illusion (Gigerenzer, 2005), two-dimensional dots with shading underneath are judged to be convex (sticking out from the plane) because the system assumes and expects the reliable environmental structure of a single source of light from above causing the shading.

***Ecological rationality implies species-specific cognition***

A direct implication of the ecological rationality framework is that cognition (including perception) will be species-specific, with sensory systems viewed as functional products of natural selection that fit each species’ behavioral needs to their environments—providing a unique interface, as Felin et al. say, between the two. Species-specific heuristics are necessary because different species operate within diverse ecological niches and use a range of core cognitive capacities. For instance, the ant *Leptothorax albipennis* has no direct way to measure the size of a candidate nest site, but this ant lays a fixed-length pheromone trail and—on a second fixed-length pass through the site—uses trail intersection rate as a heuristic estimate of its size (Hutchinson & Gigerenzer, 2005). Or consider oscars (*Astronotus ocellatus*, a territorial species of cichlid fish), which use visual cues to modulate aggression toward intruders on the basis of relative body size, exhibiting peak aggression toward dummies that are ~25% smaller than themselves (Beeching, 1992). Finally, individual differences extend similarly to judgments based on perception: Recognition is a key cue used in a variety of simple heuristics (e.g., take-the-best and the recognition heuristic; Gigerenzer, Todd, & the ABC Research Group, 1999) that can be perceived only in relation to the individual’s own past experience, as experience determines what a given individual recognizes. Although Felin et al. call for future work to account for subjective *Umwelt*ian perceptual specificity, this fits closely with extant research in ecological rationality that explicitly concerns “the subjective ecology of the organism that emerges through the interaction of its mind, body, and sensory organs with its physical environment (similar to von Uexküll’s . . . notion of *Umwelt*)” (Todd & Gigerenzer, 2012, p. 18).

Despite this study of organism- and species-level differences in perception and cognition, ecological rationality also proposes cross-species commonalities that allow us to understand some of the thinking of one species through analogies to the thinking of another. Felin et al. suggest that this implies a closeted faith in both the all-seeing eye and the “objective” nature that it perceives. But even if there were no such thing as veridical perception—no direct access to the “true” nature of reality—the subjective nature of perception is not grounds for dismissing basic assumptions of objective physical materialism. Physicist Stephen Hawking suggests that “it makes no sense to ask if [a theory] corresponds to reality, because we do not know what reality is independent of a theory” (1993, p. 44). Nothing is “truly” orange about electromagnetic radiation with wavelengths around 600 nm, and nothing is “truly” tart about citric acid—tangerines do not “look” or “taste” like anything at all—but we do not have a successful model of reality that omits physics and chemistry, so it serves no purpose to suggest that an object, which (to us) looks and tastes like a tangerine, does not have approximately measurable relative properties such as mass, position, temperature, variable reflection and absorption of electromagnetic radiation, a quantity of sugar molecules, and so on (see Hickok, 2015, and other articles from that issue for further debate). Ultimately, the “true” nature of reality is irrelevant; as long as *something* is real, and that something (whatever it is) is tied to evolutionary fitness, natural selection (blind watchmaker though it may be) can play the role of all-seeing eye, building individual organisms with adaptive responses to the environment based on all the situations that it—not any individual—has seen.

After criticizing approaches that “only make sense by arguing that there is a true, actual nature to environments” (p. 5), Felin et al. appear to proscribe basic, testable hypotheses about potential structural commonalities across organism–environment systems because such hypotheses are “not true to nature” (p. 6); yet, *truly*, natural selection binds all life on Earth to fitness constraints. Although Felin et al. rightly stress the “organism-specific factors that direct perception and attention” (p. 12), they are relatively silent regarding the often organism-general selection pressures and fitness opportunities toward which perception and attention are typically directed (e.g., collecting energy from the environment, evading predators, finding a mate, etc.). Consistent with this omission, Felin et al. charge that “[it is not possible] to identify general factors related to objects, or environmental salience or objectivity across species, . . . as what is perceived is determined by the nature of the organism itself” (p. 12). This a priori assessment is unreasonably prohibitive. Ecological rationality proposes an adaptive toolbox of specialized tools that can be used effectively in a predictable variety of settings that share common features. The toolbox approach provides a metaphorical staging point for a particular type of hypothesis: In cases in which likely overlap exists across certain species with regard to both a given goal (e.g., collecting energy) and the structure of the ecological contexts in which that goal is respectively pursued (e.g., patches of carbohydrates in the environment and the eukaryotic capacity to metabolize them), whether and how those species might also overlap in their pursuits of that goal is an empirical question and not to be dismissed out of hand.

As an example showing a fruitful application of the adaptive toolbox approach, consider search (Hills, Todd, & Goldstone, 2008). For virtually all animals that must move about through space in search of sustenance—from humans down to simple worms—dopaminergic neurons fire when those animals encounter salient and rewarding environmental stimuli (such as species-specific food rewards; Barron, Søvik, & Cornish, 2010). Note that these neurons are not triggered by unrealistically universal perceptual input, but by species- and organism-specific sensory cues (e.g., habitual learning of stimuli that predict reward for that species/individual; Graybiel, 2008). Although optimal foraging theory does assume a hypothetical “all-seeing” eye, it is the blind eye of natural selection. Neither researcher nor foraging agent knows what a “truly” optimal strategy looks like, but human researchers have the unique knowledge that every living forager on Earth has been shaped by natural selection and is likely equipped with a perception-general fitness detector that signals the presence of species-specific motivators in the environment. An estimate of how an organism could theoretically behave in order to maximize its intake while foraging (by deciding when to leave a patch and find another one) is simply a tool for researchers—a hypothesis used to predict how natural selection could have shaped an organism’s behavioral and perceptual apparatus to fit a given environmental structure.

Ecological rationality is thus a perspective that is both akin to but also more detailed than the view of “user-centric” perception that Felin et al. argue for. But even more so, ecological rationality provides a strong theoretical reason to expect that perception will be limited and specific, rather than all-seeing: simple heuristics work well exactly because they work with limited information. As Felin et al. state, “the fact that many aspects of reality are hidden is useful rather than a computational problem or lack of objectivity on the part of the organism or observer” (p. 11). The reason for this is that limited perceptual input helps avoid the problem of overfitting noisy or unimportant data, allowing simple heuristics to remain useful and robust over time. Simple heuristics reduce error from overfitting by having a bias, which can be formally treated as the bias–variance trade-off (Gigerenzer & Brighton, 2009). In fact, “a ‘veridical’ system would overwhelm the mind with a vast amount of irrelevant details” (Gigerenzer, 2005, p. 5).

To summarize, Felin et al. have put a critical feature of theorizing on the spot: the assumption of an omniscient perceiver who knows exactly what is best and can determine the optimal behavior in every problem studied. Although that may be the case in very simple tasks, such as in well-defined games, optimization is impossible in situations of uncertainty. Yet Felin et al. miss the point that Simon’s program of bounded rationality addresses just such situations wherein what is optimal is indeterminable by definition. Just as there are many visions of cognition, there are also many visions of the relationship between the mind and its environment, and many of them are similarly undermined by their visual underpinnings. Shepard’s mirror notion of the mind (with minds “reflecting” structures in the environment) and Brunswik’s mind lens model (with minds “refracting” perceived cues into internal judgments) typify such visual metaphors in which the fit between minds and environments relies upon veridical, universal perception (Todd & Gigerenzer, 2001). Ecological rationality draws instead on Herbert Simon’s nonvisual metaphor of the mind fitting to the environment like a pair of scissors. This image emphasizes how the two blades fit to each other—complementarily, not as exact mirror images—to work together and get the job done. This requires perception to provide information about the task-relevant aspects of an organism’s environment—not veridical, universal, and all-seeing, but useful, specific to the perceiver, and seeing just enough.

**Perceptual illusion, judgmental bias, and the limits of knowledge**

Barry Schwartz

Felin, Koenderink, and Krueger (2017) offer a three-part argument about research on heuristics, biases, and other violations of “rationality.” The first part is to show how much the work of Herbert Simon, Daniel Kahneman, and Amos Tversky on judgment and decision making has been modeled on accounts of visual perception. The second part is that these accounts of visual perception are based in an assumption of “inverse optics,” wherein the environment teaches the visual system how to see the world as it is. The final part is that this model of visual perception—of the “all-seeing eye”—is mistaken, that there is no one way that the visual world is. As Felin et al. put it, “the standard paradigm uses a world-to-mind, rather than a mind-to-world, model of perception that is, quite simply, not true to the nature of perception”. The implication of their argument is that research on heuristics and biases is similarly mistaken in its emphasis on error—on deviation from canonical principles of rational judgment and choice. In this comment I will attempt to elaborate on some of the authors’ points, and also to speculate on why theories of both visual perception and judgment and decision-making seem tied to normative models of seeing the world as it is (perception and judgment) and choosing in a way that is consistent with the theory of rational choice.

Kahneman (2003, 2011) is quite explicit in taking the study of visual perception as a model of judgment and choice. The all-important concept of “framing” (e.g., Kahneman & Tversky, 1984; Tversky & Kahneman, 1981) is almost explicitly modeled on visual perception, and reference-dependent choice is modeled on visual contrast effects, as is the heuristic of anchoring and adjustment. And the acronym introduced in his best-selling book (Kahneman, 2011)—WYSIATI (“what you see is all there is”) could not be more direct in its relation to visual perception. What seems to bother Felin et al. is not the relation between judgment and decision on the one hand, and visual perception on the other, but the fact that research on human judgment has focused on “error” or “bias.” And what bothers them about this focus is the use of the term “error.” Whether a judgment is an error often depends on its deviation from the norms of rational choice theory, and Felin et al. have a problem accepting these norms. Felin et al. make similar arguments about the use of the word “illusion” in research on visual perception. The term “illusion” is parasitic on the “all-seeing eye” in the same way that “error” or “bias” is parasitic on the theory of rational choice. And both normative standards are highly problematic. Felin at al. have no issue with researchers studying illusions to learn how the visual system works, as long as people do not take the word “illusion” too literally. And they feel the same way about “bias” or “error” in judgment.

In my reading, Kahneman and Tversky are less guilty of relying on the rational-choice norm than perhaps students of visual perception are of relying on the all-seeing eye. Kahneman and Tversky repeatedly stressed that it was misleading to focus too much on “error” in studying heuristics and biases, a point made repeatedly by Gigerenzer and colleagues over the years (e.g., Gigerenzer & Todd, 1999; Goldstein & Gigerenzer, 2002; Todd & Gigerenzer, 2012). Because many of these biases were surprising, they attracted attention, but what was really interesting about them was not that they were mistakes, but that they taught us something important about how the system of judgment and decision making worked. Indeed, in presenting his WYSIATI heuristic (Kahneman, 2011), Kahneman explicitly departs from the “all-seeing eye,” since he does not mean by WYSIATI that what people apprehend is literally all there is, but that what people apprehend is all that will affect their judgment and decision.

I find myself in essentially complete agreement with Felin et al. Their arguments about how visual perception works harken back to the “new look” in perception that came to prominence more than half a century ago (e.g., Bruner & Goodman, 1947; Bruner & Postman, 1947). Just as Felin et al. do, proponents of the new look very much focused on the whole organism—social and cultural background, aspirations, expectations, motives—as a key contributor to perceptual judgment. In the early days of cognitive psychology, Neisser’s (1967) landmark book similarly called our attention to “analysis by synthesis” models of cognitive processes, according to which the organism (person) played an active role in perception by taking fragments of sensory data and using them to “construct” the percept, just as a paleontologist “analyzes” a collection of dinosaur bones by constructing the whole skeleton. According to new look theorists, perception can be very much a “top-down” affair, involving active participation of the perceiver rather than passive, bottom-up registration and computation of sensory information to match physical reality.

My own work has not been about visual perception, but I have certainly questioned the normative status of rational choice theory (e.g., Keys & Schwartz, 2007; B. Schwartz, 1986, 2015). The article with Keys introduced what we called “leaky rationality.” The thrust of our argument was that the context of choice can “leak” into one’s experience with the result of that choice, often validating in experience what seem to be judgment and choice errors. This idea was stated quite explicitly by Kahneman and Tversky themselves, when they said “In [some] cases, the framing of decisions affects not only decision but experience as well. . . . In such cases, the evaluation of outcomes in the context of decisions not only anticipates experience but also molds it” (Kahneman & Tversky, 1984, p. 48).

To take just one example, we are told by rational choice theory that the “sunk cost fallacy” is indeed a fallacy—an error. If one has bought a movie ticket, but finds after 30 min that the movie is terrible, one should leave. The “rational” calculation is of the utility of sitting through the bad movie in comparison to the utility of doing almost anything else. The cost of the ticket is “sunk,” so why waste another hour of one’s life seeing the movie to the bitter end, just to get one’s money’s worth? The answer, we proposed, is that for some (or many) people, if they walk out of the movie, they will have to live with the regret of having wasted money, an emotion they can avoid by sitting through the movie until the end. For a person like this, is the sunk cost fallacy still a fallacy? If the aim of a decision is to enhance utility—a subjective quantity—it is hard to know from what stance to criticize someone who quite reasonably acts so as to avoid regret, and thus to enhance utility. In an underappreciated study, Frisch (1993) gave participants problems of the sort that typically elicited “errors” in Kahneman and Tversky’s research. The problems involved asking participants questions with identical underlying structures but different surface forms. The typical Kahneman and Tversky finding was that people were unable to see through surface form to underlying structure, so that they routinely gave different answers to what were, in some sense, two versions of the same questions. Frisch then confronted people with the two versions of the problems on which they had given different answers and asked whether, examining them side by side, the people still thought the problems were different. In virtually every case, the majority of people who had given different answers to formally identical but superficially different versions of a given problem insisted that they were not two versions of the same thing.

So, in line with Felin et al., it seems sensible to me to view visual illusions and judgmental biases as data, not errors. And Felin et al. could have gone farther. Sometimes, distorted perceptions of the visual world are actually quite beneficial. Lateral inhibition enhances boundaries between objects. Sensory adaptation makes it easier to detect novelty. Attention, which suppresses much of what is going on in the visual field, enables us to process what we are attending to in greater depth and detail. Lateral inhibition, adaptation, and attention are all distortions. But we could not get through a day without them.

Similarly, in the domain of judgment and decision, people seem to fail to appreciate that money is fungible, and divide expenditures and receipts into different mental accounts. They pay 14% interest on credit card balances while still putting $25/week into a savings account that earns 1%. The money that goes into the bank is in a different “mental account” (perhaps a “vacation,” “retirement, “ or “childrens’ college” account) than the money they use to pay off credit-card debt. In a simple, laboratory setting, such examples of mental accounting seem foolish. But in the complexity of the natural world, dividing income and outflow into accounts may be the only way to make sense of anything. How long would it take to decide what to do with a $1,000 graduation gift if one put everything one could possibly do with $1,000 on the table as a possibility? Thus, as Thaler (1999) pointed out, not only does mental accounting matter, but it actually helps us organize almost unimaginable complexity into manageable chunks. People keep mental accounts, and there is no plausible normative theory of how they *should* keep mental accounts. Indeed, it seems clear to me that the rational-choice norm in judgment and decision making is far more dubious than the all-seeing eye norm in visual perception. One can imagine using physical measuring instruments to determine what is “really” out there as a benchmark against which to compare what people see. In contrast, rational-choice theory is a norm for making decisions that serve “utility” or “preference,” not wealth. But utility is a fundamentally subjective concept. Utility is person-dependent. As Stigler and Becker (1947) famously said, “*de gustibus non est disputandum*” (there is no accounting for taste).

In the face of so much evidence that the “all-seeing eye” is not a useful normative model of visual perception and the “rational economic agent” is not a useful normative model of judgment and decision, it is worth asking why researchers cling so tenaciously to these models. I propose that the answer may lie in an argument made by Fodor (1983). Thirty-five years ago, Fodor published a highly influential and controversial book, *The Modularity of Mind* (and see M. F. Schwartz & Schwartz, 1984, for a detailed discussion). In the book, Fodor divided the mind into three sectors: sensory processors, the “central system,” and what he called “input modules.” The job of the input modules was to take the outputs from sensory processors and turn them into a form that could be utilized by the central system.

Fodor (1983) made a variety of bold claims about the nature of input modules and about modularity more generally. They generated a great deal of research aimed at identifying modules and characterizing what kinds of information they could exploit and what kinds were opaque to them. Much less attention was devoted to Fodor’s characterization of central systems, but as bold as Fodor’s claims were about modules, his claims about central systems were even bolder. The central system is what we normally have in mind when we talk about deliberation, reason, and thinking. It is the part of the cognitive apparatus that we are using when we are consciously and deliberately trying to make sense of something. According to Fodor, the key difference between central systems and modules is that whereas modules are “informationally encapsulated” (they can only respond to certain types of information, even when other information might be relevant to their information-processing tasks), central systems are *not* informationally encapsulated. Anything might be relevant to the interpretive problem at hand, and thus the central system is wide open to all kinds of influences. But the problem, according to Fodor, is that for just this reason, we cannot have a science of central systems. Because context always matters, and because every context is in some ways unique, there are no lawful generalizations about central systems to be had, except, perhaps, for generalizations so abstract that they yield little in the way of predictions in specific situations.

According to Fodor (1983), for a science to make progress, practitioners must be able to “carve nature at its joints”—that is, scientists must be able to divide their domain of study into meaningful, well-behaved chunks. Modules do this. But the central system, Fodor argues, has no joints. The example that Fodor uses to make his case is the development of science itself. Scientific activity is paradigmatically central-system activity. But, Fodor observes, despite centuries of effort, we have no science of science. Indeed, he suggests, we *can* have no science of science. This is partly because when it comes to a scientific understanding of a domain, anything might be relevant (a characteristic he calls “isotropic”). And it is partly because, as we have known since Quine (1951; with a significant contribution by Kuhn, 1962), the notion that we can use pristine data to test theories is naïve. Theories tell us what counts as data, a characteristic of the scientific enterprise that Fodor refers to as “Quinean.”

The argument is not that scientific advance is random or capricious (though sometimes it might be). It is that scientific advance can be understood with historical analysis, and not with ahistorical, law-like generalizations. Historians help us make sense of the past, whether or not their accounts enable us to predict the future. So, according to Fodor, whereas it might be possible to develop a history of central systems, just as we have a history of science, we can’t expect to develop a science of central systems.

In my view, a substantial appeal of the “all-seeing eye” and “inverse optics” in visual perception, and of the rational-choice model in decision making, is that “objective” stances like these enable us to develop a science. If Felin et al. are correct that these approaches ignore the key role of the organism, both in perception and in judgment and decision making, then we are entering the domain of what Fodor called *central systems*. Anything might be relevant to a perceptual judgment, a valuation, a probability estimate, or a choice. Past experience, current context, attentional focus, judgmental anchors, and individual motives and desires might each play a role in what people see, what they want, and what they choose. Felin et al. speak with great enthusiasm (which I share) about Uexküll’s (2010) appreciation of the *Umwelt—*the subjective rather than the objective world. The city park is a different place for the foraging squirrel than for the strolling urbanite. Perception is subject-relative. But can’t there be a science of *Umwelten*—of species-specific perceptual filters that are attuned to the adaptation problems faced by each species? I assume the answer to this question is yes, as has admirably been demonstrated by a century of research in ethology. But it is crucial to realize that Uexküll was writing about filters that were both species-specific *and* species-typical—all hungry squirrels would see the same park. In effect, to use Fodor’s terminology, Uexküll was writing about input modules. When it comes to human beings, species typicality is lost, at least if Fodor is right about central systems. Each of us brings a different history and a different agenda to a particular situation—perceptual or judgmental—with the result that, as with science, we can perhaps make sense of things after the fact, but we can’t expect to find law-like generalizations that will enable us to predict before the fact. Felin et al. may be right about how visual perception (and judgment and decision) actually work, but they fail to appreciate, I think, how high the stakes are in accepting their view of the world. They write enthusiastically about Uexküll, perhaps without fully appreciating that his species-typical subjectivity is different from their own claims about individually idiosyncratic subjectivity.

Laboratory settings are created to simplify the complex, to establish precise control of conditions, and to keep out the “extraneous.” The presumption here is that successful laboratory settings will reveal pieces of what might be a complex process clearly and unambiguously in a way that would never be possible outside the laboratory. The analytic tool of the laboratory experiment has probably done more to enable scientific progress than any other aspect of scientific activity (see Horton, 1967, for an argument that the experiment is what most distinguishes scientific thought from folk–traditional thought). The presumption that justifies the experimental approach is that if one can take complex things apart in the laboratory, it is a small step (though one rarely taken in practice) to put them back together outside the laboratory. But if my interpretation of Felin et al. is correct, the task of putting things back together is neither small nor unproblematic. As Bennis, Medin, and Bartels (2010) put it, the world of the laboratory is a “closed system,” whereas the world we actually live in is an “open system.” What works in a closed system may not work in an open one, and what seems like an error in a closed system may be the best people can do in an open one. Consider again, for example, the effects of frames and mental accounts in decision making (Kahneman & Tversky, 1984; Thaler, 1999; Tversky & Kahneman, 1981). Framing and mental-accounting effects are often regarded as cognitive shortcomings, as “mistakes.” But rational decision making might be essentially impossible without such frames and accounts (B. Schwartz, 1986). Decision frames come into their own in open systems. If Fodor’s discussion of what central systems are and how they operate is roughly correct, then we will never understand them by creating an environment, like the laboratory, that distorts their fundamental nature.

The possibilities I raise here could apply quite broadly in psychology. As Gergen (1973) pointed out many years ago, in an article aptly titled “Social Psychology as History,” many of the phenomena that psychologists are most interested in understanding might be largely the province of Quinean and isotropic central systems.

In this time of “fake news” and “alternative truth,” I do not want to be understood as suggesting, nihilistically, that we can never really know anything. We can know plenty, and science has found out a great deal about the things it studies, if not so much about itself. A key reason for the progress that science makes, I believe, is not that science trains its practitioners to see the world as it is. No, I suspect that scientists are just as prone to effects of framing, context, and aspirations as anyone else. What makes science capable of real progress, I think, is that it is public. The community of scientists, in public conversation, corrects the “biases” and “illusions” that each of them has as individuals. Public science makes progress by means of what has been called “the wisdom of crowds” (Surowiecki, 2004)—in this case, highly trained crowds. Like proverbial blind men feeling around the parts of an elephant, scientists, like the rest of us, if not blind, are at least a little myopic. They rely on their colleagues to save them from embarrassment or worse.

In his thoughtful book, *Time’s Arrow, Time’s Cycle*, Stephen J. Gould (1987) distinguished between processes in nature that are repeatable (“time’s cycle”) and processes that are historical (“time’s arrow”). Gould regarded the theory of evolution as the paradigm case of a science that is essentially historical. As an enthusiastic contributor to geology and evolutionary science, Gould was hardly suggesting that because it was historical, evolutionary theory could not be scientific. What he *was* suggesting, however, was that to capture evolutionary processes, we needed a different model of science than the one handed down by physics. We needed explanation, not prediction. Exactly the same might be true when it comes to understanding the operation of the central system. And this is what I take to be the broadest implication of the Felin et al. argument. Where does that leave psychology? Not like physics, perhaps, but the science of psychology could do much worse than ending up as a science with the explanatory power of the theory of evolution.

**Perceiving rationality correctly**

Keith Stanovich

No important conclusions about rational thought depend on issues of perceptual theory at the level dealt with in Felin, Koenderink, and Krueger’s (2017) essay. It is true that several important theorists in the heuristics-and-biases literature have used analogies with perception to facilitate the understanding of cognitive biases. The perceptual examples used by Kahneman and others were used to highlight certain cognitive biases, but the implications of the heuristics-and-biases work for the study of rationality in no way depend on any theory of the visual illusions that were used. The arguments about human decision making that have formed the heart of the Great Rationality Debate (GRD) in cognitive science (Cohen, 1981; Stanovich, 1999; Stein, 1996; Tetlock & Mellers, 2002) stand or fall on their own, independent of developments at this extremely abstract level of perceptual theory.

The authors themselves, on page 14, say that “our arguments about perception may seem abstract and perhaps far removed from practical concerns about the study of rationality.” I couldn’t agree more. These arguments about perception are indeed abstract. They are indeed far removed from practical concerns about the study of rationality. This far-fetched link between the literature on rational thinking and the literature at an abstract level of perceptual theory seems to be employed here only to provide a seemingly new rationale for the authors to launch a largely redundant critique of the heuristics-and-biases literature.

It is a redundant critique because many of these criticisms have arisen and been dealt with throughout the last three decades of work on heuristics-and-biases tasks and the critiques of them. The Felin, Koenderink, and Krueger essay is backward-looking in that it revives old debates that have been resolved for some time now. The answers to virtually all of these criticisms are contained in the GRD synthesis that has been used in the field for over a decade.

That synthesis derives from works well into their second decade now, including, in chronological order: Stanovich (1999, 2004), Stanovich and West (2000), Kahneman and Frederick (2002), and Samuels and Stich (2004). The synthesis relies on contemporary dual-process theory (Evans & Stanovich, 2013; Kahneman, 2011). It also relies on two decades worth of work on individual differences in rational thought (Stanovich, West, & Toplak, 2016).

The synthesis follows from interpreting the responses primed by Type 1 and Type 2 processing as reflecting conflicts between two different types of optimization—fitness maximization at the subpersonal genetic level, and utility maximization at the personal level. The synthesis acknowledges a point that the critics of the heuristics-and-biases literature have stressed: that evolutionary psychologists have often shown that the adaptive response on a particular task is the modal response on the task—the one that most subjects give. However, that data pattern must be reconciled with another finding often obtained: that lower cognitive ability is often associated with the response deemed adaptive on an evolutionary analysis (Stanovich, 1999; Stanovich & West, 2000; Stanovich et al., 2016). The synthesis of the GRD referred to above argues that the evolutionary interpretations do not impeach the position of heuristics-and-biases researchers that the alternative response given by the minority of (more cognitively able) subjects is rational at the level of the individual. Subjects of higher analytic intelligence are simply more prone to override Type 1 processing in order to produce responses that are epistemically and instrumentally rational.

A point repeatedly made from within the GRD consensus position is that both Type 1 and Type 2 processing lead to rational responses most of the time. Thus, most of the time the outputs from the two systems are in sync, and there is no conflict. The controversy that spawned the GRD from the beginning was the invention of heuristics-and-biases tasks that primed two different responses, one from each of the systems. The assumption behind the current GRD synthesis is that the statistical distributions of the types of goals being pursued by Type 1 and Type 2 processing are different. The greater evolutionary age of some of the mechanisms underlying Type 1 processing accounts for why such processing more closely tracks ancient evolutionary goals (i.e., the genes’ goals) than does Type 2 processing, which instantiates a more flexible goal hierarchy that is oriented toward maximizing overall goal satisfaction at the level of the whole organism. Because Type 2 processing is more attuned to the person’s needs as a coherent organism than is Type 1 processing, in the minority of cases in which the outputs of the two systems conflict, people will be better off if they can accomplish a system override of the Type-1-triggered output (Stanovich, 2004). The response triggered by System 2 is the better statistical bet in such situations, and that is why it correlates with cognitive ability.

What I am calling the GRD synthesis reconciles most of the debates between the heuristics-and-biases researchers and their critics. The GRD synthesis has been around for quite some time now and has been reiterated in the literature many times. This is why it is surprising to see some of the same old shopworn issues coming up again in this essay. The authors keep reiterating the point that heuristics (Type 1 processing) are useful most of the time, often give the normative response, and that they are adaptively efficient (“many of the seeming biases have heuristic value and lead to better judgments and outcomes,” p. 14; “the vast amount of decision making that humans get right receives little attention,” p. 16; “apparent biases might be seen as rational and adaptive heuristics,” p. 16). But, as I noted previously, dual-process theorists have been at pains to state that Type 1 processing is efficacious most of the time and that reliance on Type 1 processing does not always lead to error. Evans and I pointed out that the equation of Type 1 processes with all bad thinking and Type 2 processes with correct responding is the most persistent fallacy in the history of dual-process theory (now reaching its 40th anniversary; Posner & Snyder, 1975; Shiffrin & Schneider, 1977; Wason & Evans, 1975). Likewise, the early originators of the heuristics-and-biases research tradition consistently reiterated that Type 1 processing modes often lead to normative responding and efficient task performance (Kahneman, 2000, 2011). The GRD synthesis long ago gave up this fallacy, so it is surprising to see it reiterated so often here, or used to create a straw man in statements like “the human susceptibility to priming and sensitivity to salient cues is not prima facie evidence of irrationality” (p. 16). Of *course* System 1 priming is not prima facie evidence of irrationality! No dual-process theorist has ever made this claim. All of the early dual-process theorists (e.g., Posner and Shiffrin; see above) assumed that priming in the human brain was efficacious, as have all subsequent theorists.

Other critiques in this essay likewise seem to take us backward to old issues long resolved. The end of the essay reads like a Panglossian litany. In the GRD literature, a Panglossian is the type of theorist who tries to close every gap between the descriptive and the normative that is revealed by empirical research (Stanovich, 1999, 2004; Stein, 1996). Such a theorist has many options. First, instances of reasoning might depart from normative standards due to performance errors (temporary lapses of attention and other sporadic information-processing mishaps). Second, computational limitations may prevent the normative response. Third, in interpreting performance, we might be applying the wrong normative model to the task. Alternatively, we may be applying the correct normative model to the problem as set, but the subject might have construed the problem differently and be providing the normatively appropriate answer to a different problem.

All of these (random performance errors, computational limitations, incorrect norm application, and alternative problem construal) are alternative explanations that avoid ascribing subpar rationality to a response—and they have all been extensively discussed in the literature. But numerous theorists have warned that it is all too easy to use the alternative interpretations in an unprincipled, cherry-picked way that makes Panglossianism unfalsifiable. Rips (1994) warned that “a determined skeptic can usually explain away any instance of what seems at first to be a logical mistake” (p. 393). Kahneman (1981) argued that Panglossians seem to recognize only two categories of errors, “pardonable errors by subjects and unpardonable ones by psychologists” (p. 340). Referring to the four classes of alternative explanation discussed above—random performance errors, computational limitations, alternative problem construal, and incorrect norm application—Kahneman noted that Panglossians have “a handy kit of defenses that may be used if subjects are accused of errors: temporary insanity, a difficult childhood, entrapment, or judicial mistakes—one of them will surely work, and will restore the presumption of rationality” (p. 340).

In short, the toolkit of the Panglossian is too large and too prone to be applied in an unprincipled manner. For years, theorists have pointed to the need for principled constraints on the alternative explanations of normative/descriptive discrepancies. Our own work on individual differences (Stanovich et al., 2016; Toplak, West, & Stanovich, 2011; West, Toplak, & Stanovich, 2008) was originally motivated by the need to provide such principled constraints (Stanovich & West, 1998, 2000). Yet the critiques in the last three pages of this essay simply proceed as if these debates had not occurred and already generated a research literature—almost as if we were back in the time of Cohen (1981), at the root of the GRD. As if this were a new insight, we are repeatedly warned about alternative construals:

Furthermore, this alternative theory needs to recognize that many of the simplistic tests of rationality omit important contextual information and also do not recognize that even simple stimuli, cues, and primes can be interpreted in many different ways. (p. 14)

There is a large variety of stimuli that could be pointed to (and proven) but missed by human subjects in the lab or in the wild. But these types of findings can be interpreted in a number of different ways. (p. 15)

As if this were a new insight, we are repeatedly warned about alternative norms:

granting scientists themselves an all-seeing position—against which human decision making is measured. The conventional and even ritualistic use of this null hypothesis has endowed it a normative force. Yet, repeated rejections of this null hypothesis are of limited interest or concern when the normative status of the theory is itself questionable. (p. 14)

Visual illusions reveal that multiple responses, or ways of seeing, are equally rational and plausible. (p. 16)

we argue that even simple stimuli are characterized by indeterminacy and ambiguity. Perception is multistable, as almost any percept or physical stimulus—even something as simple as color or luminance (Koenderink, 2010)—is prone to carry some irreducible ambiguity and is susceptible to multiple different interpretations. (p. 16)

Unmentioned are the constraints on the alternative construals and alternative norms that have been empirically investigated in the years since Cohen (1981). Also unmentioned is a fact that embarrasses many of these Panglossian critiques: Most subjects in heuristics-and-biases experiments retrospectively endorse the Bayesian and subjective expected utility norms that they violate. That is, after responding—usually after failing to override the response that comes naturally (Kahneman, 2003)—subjects choose the correct norm that they were led to violate (Kahneman & Tversky, 1982; Shafir, 1993, 1998; Shafir & Tversky, 1995; Thaler, 1987). When shown the multiple norms that Felin et al. stress repeatedly, subjects are more likely to endorse the Bayesian norm than alternatives (Stanovich & West, 1999). In introducing the collection of Amos Tversky’s writings, Shafir (2003) stressed this very point: “The research showed that people’s judgments often violate basic normative principles. At the same time, it showed that they exhibit sensitivity to these principles’ normative appeal” (p. x). For example, Koehler and James (2009) found that nonnormative “probability matchers rate an alternative strategy (maximizing) as superior when it is described to them” (p. 123). In short, when presented with a rational-choice axiom that they have just violated in a choice situation, most subjects will actually endorse the axiom. If people nevertheless make irrational choices despite consciously endorsing rational principles, this suggests that the ultimate cause of the irrational choices might reside in Type 1 processing and the miserly tendency not to override it with Type 2 processing.

Consider framing effects and preference reversals, two of the most researched ways of demonstrating deviations from instrumental rationality (Kahneman, 2011; Lichtenstein & Slovic, 2006). In such problems, subjects often agree in postexperimental interviews that the two versions are identical and that they should not be affected by the wording. In short, preference reversals or framing effects do not represent alternative contextualizations that subjects *want* to have. Instead, such alternative construals represent *mental contamination* (Wilson & Brekke, 1994) that the subjects would choose to avoid.

The issue of postexperimental endorsement is just one way of employing the understanding/acceptance assumption in the GRD—that more reflective and engaged reasoners are more likely to affirm the appropriate normative model for a particular situation (Slovic & Tversky, 1974; Stanovich & West, 1999). Subjects actively reflecting on the norms are more likely to indicate the norms they want to follow. Likewise, individuals with cognitive/personality characteristics more conducive to deeper understanding are more accepting of the appropriate normative principles for a particular problem. That is the result of the individual-differences work I mentioned above.

The authors keep reiterating that the extant literature emphasizes bias too much. In fact, there is no way to tell whether there has been too much or too little emphasis on bias. To know that, someone would have to know the exact distribution of benign and hostile environments a person must operate in and the exact costs and benefits of defaulting to Type 1 processing in every single environment (talk about omniscience!). The point (extensively discussed by Kahneman, 2011) is that an attribute-substituting System 1 and a lazy System 2 can combine to yield rational behavior in benign environments but yield seriously suboptimal behavior in hostile environments. A benign environment is an environment that contains useful cues that, via practice, have been well represented in System 1. Additionally, for an environment to be classified as benign, it must not contain other individuals who will adjust their behavior to exploit those relying only on System 1 heuristics. In contrast, a hostile environment for Type 1 processing is one in which none of the available cues are usable by System 1 (causing the substitution of an attribute only weakly correlated with the true target). Another way that an environment can turn hostile is if other agents discern the simple cues that are triggering the cognitive miser’s System 1—and the other agents start to arrange the cues for their own advantage (e.g., in advertisements or the deliberate design of supermarket floor space to maximize revenue).

The Meliorist (see Stanovich, 1999, 2004) supporters of the heuristics-and-biases approach see that approach as ideally suited to studying cognition in the modern world. The beguiling (but wrong) intuitive response in heuristics-and-biases tasks is viewed as a strength and not a weakness. It is a design feature, not a bug. Why? Because the modern world is, in many ways, becoming hostile for individuals who rely solely on Type 1 processing. The Panglossian theorists have shown us that many reasoning errors might have an evolutionary or adaptive basis. But the Meliorist perspective on this is that the modern world is increasingly changing so as to render those responses less than instrumentally rational for an individual. Einhorn and Hogarth (1981) long ago made the telling point that “in a rapidly changing world it is unclear what the relevant natural ecology will be. Thus, although the laboratory may be an unfamiliar environment, lack of ability to perform well in unfamiliar situations takes on added importance” (p. 82).

Critics of the abstract content of most laboratory tasks and standardized tests have been misguided on this very point. Evolutionary psychologists have singularly failed to understand the implications of Einhorn and Hogarth’s warning. They regularly bemoan the “abstract” problems and tasks in the heuristics-and-biases literature and imply that since these tasks are not like “real life,” we need not worry that people do poorly on them. The issue is that, ironically, the argument that the laboratory tasks and tests are not like “real life” is becoming less and less true. “Life,” in fact, *is becoming more like the tests!* Try arguing with your health insurer about a disallowed medical procedure, for example. The social context, the idiosyncrasies of individual experience, the personal narrative—the “natural” aspects of Type 1 processing—all are abstracted away as the representatives of modernist technological-based services attempt to “apply the rules.”

Unfortunately, the modern world tends to create situations in which the default values of evolutionarily adapted cognitive systems are not optimal. Modern technological societies continually spawn situations in which humans must decontextualize information—where they must deal abstractly and in a depersonalized manner with information rather than in the context-specific way of the Type 1 processing modules discussed by evolutionary psychologists. The abstract tasks studied by the heuristics-and-biases researchers often accurately capture this real-life conflict. Likewise, market economies contain agents who will exploit automatic Type 1 responding for profit (better buy that “extended warranty” on a $150 electronic device!). This again puts a premium on overriding Type 1 responses that will be exploited by others in a market economy. The commercial environment of a modern city is not a benign environment for a cognitive miser. To the extent that modern society increasingly requires the Type 1 computational biases to be overridden, then Type 2 overrides will be more essential to personal well-being.

Evolutionary psychologists have tended to minimize the importance of the requirements for decontextualizing and abstraction in modern life (the “unnaturalness” of the modern world that in fact matches the “unnaturalness” of many laboratory tasks!). For example, Tooby and Cosmides (1992) use the example of how our color constancy mechanisms fail under modern sodium vapor lamps; the authors warn that “attempting to understand color constancy mechanisms under such unnatural illumination would have been a major impediment to progress” (p. 73)—a fair enough point. But what it misses is that if the modern world were structured such that making color judgments under sodium lights was critical to one’s well-being, then this would be troublesome for us because our evolutionary mechanisms have not naturally equipped us for this. In fact, humans in the modern world are in just this situation vis-à-vis the mechanisms needed for fully rational action in highly industrialized and bureaucratized societies.

Thus, the longstanding debate between the Panglossians and the Meliorists can be viewed as an issue of figure and ground reversal. It is possible to accept most of the conclusions of the work of Panglossian theorists but to draw completely different morals from them. For example, evolutionary psychologists want to celebrate the astonishing job that evolution did in adapting the human cognitive apparatus to the Pleistocene environment. Certainly they are right to do so. But at the same time, it is not inconsistent for a person to be horrified that a multimillion dollar advertising industry is in part predicated on creating stimuli that will trigger Type 1 processing heuristics that many of us will not have the disposition to override. To Meliorists, it is no great consolation that the heuristics so triggered were evolutionarily adaptive in their day.

**Cues, minds, and equilibria: Responses and extensions**

Teppo Felin, Jan Koenderink, Joachim Krueger

We are thrilled to receive thoughtful commentaries on our article from prominent scholars in psychology, cognitive science, decision science, and biology. The commentaries range from highly critical to broadly supportive. We welcome the opportunity to respond to these comments and to highlight extensions and implications of our all-seeing eye argument as it applies to rationality, perception, and cognition.

Space considerations unfortunately prohibit us from addressing the commentaries point by point. Thus, we will largely focus on the commentaries that raise fundamental concerns and counterexamples to our argument (Chater & Oaksford, 2017; Funder, 2017; Gigerenzer, 2017; Nordli, Todd, & Gigerenzer, 2017; Stanovich, 2017). However, we also make passing reference to the commentaries more favorable to our original argument (Noble, 2017; Schwartz, 2017). Our response is organized around three fundamental issues—namely, (1) the problem of cues; (2) what is the question?; and (3) equilibria, $500 bills, and the axioms of rationality.

***The problem of cues***

Several commentaries argue that we either take our rationality and all-seeing eye argument too far or that the argument does not apply to their particular conception of rationality, cognition, or judgment. Funder (2017), for example, points to the situation-construal literature and argues that situational analysis in fact allows scientists to objectively study perception—thus challenging our all-seeing eye argument. Gigerenzer and colleagues (Gigerenzer, 2017; Nordli et al., 2017) argue that the all-seeing eye assumption does not pertain to the literature on heuristics and ecological rationality. We respectfully disagree with these views.

A straightforward way to illustrate how the all-seeing eye plagues both the situation construal and the heuristics or ecological rationality literatures is to point to the theoretical primacy that is placed on “cues.” Cue-focused approaches necessarily imply an all-seeing eye. The focus on cues within these two theoretical traditions—though other cognitive and psychological theories could also be used as examples—makes them *theories of perception*, as well. As we discussed in our original article, most theories of rationality and cognition tend to feature implicit meta-theories about perception and observation, and thus the underlying perceptual assumptions deserve careful scrutiny. We first discuss the situation-construal literature, in conjunction with Funder’s commentary, and then discuss Gigerenzer and colleagues’ commentaries and the particular emphases they place on cues, heuristics, and ecological rationality.

Funder and colleagues (e.g., Funder, 2016) build their model of situation construal on a general “model of situation *perception*” (see Fig. 1 and Table 2 of Rauthmann et al., 2014, pp. 679, 686). The perceptual focus is evident in the focus on cues: The word “cue” is mentioned 92 times in a recent article introducing a taxonomy of situations (Rauthmann et al., 2014). What, then, are cues? For Funder and colleagues “situation cues are physical or objective elements that comprise the environment. They can be objectively measured and quantified” (Rauthmann et al., 2014, p. 680; cf. Funder, 2016). Cues represent the “composition of the situation” (Rauthmann, Sherman, & Funder, 2015, p. 364)—the ecology or environment (cf. Pervin, 1978)—and include the following: “(a) persons and interactions (Who?); (b) objects, events, and activities (What?); and (c) spatial location (Where?)” (2014, p. 679).^1^ Funder argues that a situation can be captured from the bottom up, by surveying the observers or situational participants themselves and then using their “consensus”—that is, the averaged, modal or aggregate statistical responses (e.g., rank-order judgments of what the situation is about)—to arrive at the objective nature of the situation. The argument is that the collective responses of “socially competent observers” (Funder, 2016) will somehow reveal the objective nature of the situation.

The situation-construal argument is in fact an excellent example of the all-seeing-eye argument—the type of camera-like conception of perception and reality that we were concerned about in our original article. In the case of situation judgment, however, the all-seeing-ness is constructed not in a top-down (though top-down mechanisms are also allowed) but in a bottom-up manner, from individual observations and their aggregation. Individuals serve as observers—essentially, sensors, or recorders—of cues in their environment, from which the objective situation is constructed. In other words, if enough eyes are on it, the world can be adequately, if not exhaustively, captured and represented. This is an instance of inverse optics (Marr, 1982) and an example of the world-to-mind mapping we discussed in the target article.

This background is important, because in his commentary Funder uses the social-perception version of the inverse-optics logic to dispute our interpretation of the gorilla experiment (Simons & Chabris, 1999). Funder argues (p. 18) that the reality of the gorilla scene can “in principle” be “visually identified” by the subjects or a “detached observer.” He recognizes the challenge of capturing the scene completely, but nonetheless claims that “it can be done.” Funder suggests that the fact that we are able to, in our original article, point to any number of potential observations in the scene means that it is doable. He recognizes that some things are outside of human perception, but argues that “the portion of the environment susceptible to human observation is, for human purposes, a sufficient definition of reality.” Funder even argues that “to conclude otherwise is to undermine the foundations of science itself”. We will show, however, that no visual scene or situation can be exhausted in terms of its potential descriptions and representations, even that which sits within the human visual spectrum. We consider this to be a logical and scientific verity. Physical structure is always just that, structure, which is meaningless without some mechanism for creating salience or awareness.

Before providing examples, we must address a fundamental misunderstanding evident in Funder’s commentary. He argues that our all-seeing-eye argument somehow suggests “alternative facts” and that we’ve “give[n] up the attempt to seek truth” (p. 19). Funder misinterprets us to be saying that *nothing* can objectively be said—or that anything can be said—about a given visual scene or situation; or that some descriptions can’t be privileged over others. Our precise point is the *opposite*, which is that a near infinite variety of things can objectively be said about or “read into” a scene or situation, and in fact many of these descriptions, *depending on the problem and question at hand* (see the next section, titled What Is the Question?), can be equally objective, but *also* contradictory. No amount of observation or aggregation of cues or observers, as Funder would have it—if theory-independent—will yield “*the* right” answer about a visual scene or situation.

Situations—just like visual scenes—are teeming with latent cues and possible realities, outstripping our ability to capture them in any conclusive way. To demand that we ought to be able to do this is the all-seeing eye argument. Suppose we surveyed the six actors involved in the staging of the Simons–Chabris (1999) gorilla experiment (passing the basketball to each other), along with surveying some number of external observers, to capture the objective nature of the situation. The literature that Funder points us toward argues that if we array observers to look at a scene or situation—that is, observers who are *in situ* (within the situation), *juxta situm* (around the situation), and *ex situ* (outside the situation)—then from this we can coalesce the true, objective nature of what is happening (see Rauthmann et al., 2015). The varied observers are treated as camera-like recorders and sensors, and their aggregate observations, perceptions, and construals will generate the truth. We might also use Funder’s taxonomy and catalogue of situational cues, persons, and events to question the subjects (Rauthmann et al., 2014). The problem is the sheer volume of possible cues that one could attend to. To deal with this problem, Funder’s approach uses forced ranking as a mechanism for getting subjects to commit to *the* situation. However, the cues that any one observer may attend to are arbitrary, or perhaps simply primed through questions. But again, an indefinite number of cues are available, each of which could, in theory, be attended to. It is impossible to attend to and process all of them. More importantly, how specifically do subjects know what is relevant? Even if observers or participants happen to attend to the same visual cues, these could legitimately be “read” and interpreted in any number of ways. Not “interpreted” in any postmodern sense, but simply in the sense that these visual cues or behaviors could mean different things. No aggregate questionnaire, impartial observation, recording, or statistical procedure can yield *one*, true objective scene or situation.

To informally highlight this: Imagine that two of the participants lightly bump into each other in the gorilla clip. This illustrates a number of issues. First, whether the other participants or external observers themselves actually “register” or see this, is important—for it to show up in Funder’s aggregate, composite characterization of the situation. But let’s assume they do. The second problem is that this bump may or may not be relevant. And third, the set of potential interpretations of the bump could be varied, leading to different construals, which, again, may or may not be captured in the *overall* nature of the situation. The bump may have, objectively and truly, been some form of bullying, or it may have been some form of joking or flirting—or just an accident. The same could be said for any number of other cues in the clip, such as the visual expressions or interactional glances of the participants. Anything could be relevant (and “obvious,” like the gorilla), and any one thing could legitimately be read a number of different ways. The important point is that the cues themselves don’t somehow signal and tell us whether they are relevant or not, and what they mean.

Funder realizes that cues are mere “raw input” and need interpretation and processing. But no mechanism of salience and awareness is given. The problem is that the situations literature that Funder’s commentary points us toward is unavoidably also a theory of perception, though one that suffers from all the problems of the all-seeing eye, because the approach features no explicit theory of salience or awareness.

In their commentaries, Gigerenzer and colleagues (Gigerenzer, 2017; Nordli et al., 2017) claim that “ecological rationality needs no all-seeing eye” However, the strong focus on cues in this literature suggests otherwise. To illustrate, in a highly cited review of the heuristics and ecological rationality literature (Gigerenzer & Gaissmaier, 2011; also see Dhami, Hertwig, & Hoffrage, 2004; Luan, Schooler, & Gigerenzer, 2011) the word cue appears 92 times in the body of the article.^2^ Gigerenzer and Gaissmaier specifically discuss cues in varied ways, including *cue weighting*, *the number of cues*, *the correlation of cues*, *cue validity*, *contradictory cues*, *cue addition*, *cue cleverness*, *the search through cues*, *positive cues*, *cue value*, *first cue*, *top cue*, *discriminant cues*, *cue ordering*, *cue redundancy*, *cue correlation*, *cue integration*, *cue combination*, *cue growth*, *cue favoring*, *relevant cues*, and so forth. Of course, the fact that cues are frequently mentioned isn’t any kind of intellectual argument against this literature. However, it does place the arguments about heuristics squarely into the domain of perception—and we argue, the all-seeing eye.

The literature on ecological rationality argues that in the presence of overwhelming amounts of environmental information (cf. Simon, 1990), humans and other organisms use heuristics to focus on what is essential and useful. A heuristic is defined as anything that allows organisms to—adaptively and frugally—process and attend to the right cues and stimuli in their environments (cf. Shah & Oppenheimer, 2008), and to ignore other cues and information (Gigerenzer & Gaissmaier, 2011).

Our concern with heuristics, and with ecological rationality in general, is that there is no clear theory about *what* a cue (or information) is and *why* certain cues might be selected, attended to, and found to be “valid,” “weighted,” or “ordered” in certain ways. Cues appear to just appear. From our perspective, the focus on the right (or useful) cues is certainly an improvement over psychological and behavioral models that emphasize veridicality and some form of inherent cue salience (e.g., Kahneman’s “natural assessments”; see also Schwartz’s discussion of “what you see is all there is”). But the model of ecological rationality fails to provide a theory of salience itself—that is, why organisms might be aware of or care about particular cues. The right cues are simply given. Saying that attention to the right cues represents a heuristic does not explain *why* these cues are selected. Thus, we might provocatively conclude that heuristics merely give the perceptual problem a new name, rather than explaining it.

Gigerenzer and colleagues acknowledge that species-specific factors are important (Gigerenzer, 2017; Nordli et al., 2017), and presumably these factors play a role in cue salience and selection. But these factors appear to be immaterial to their theory, as illustrated by their generic gaze heuristic and recognition heuristics (Gigerenzer, 2003; Gigerenzer & Goldstein, 2011). Gigerenzer et al. postulate that heuristics have evolutionary origins, thus suggesting that *very* long-run, universal mechanisms (such as natural selection) determine which cues organisms attend to. This evolutionary argument allows them to make *general* claims; as they say, “ecological rationality proposes cross-species commonalities” and “organism-*general* selection pressures” (Nordli et al., 2017, p. 31). We concur with them that all organisms exist in environments and are prone to selective pressures. But we consider it essential to consider and study the particular environments (or *Umwelten*) in which species operate. Organism-*general* models are ill-suited to explain the objects of interest, salience, and contents of awareness, with life or death consequences for many organisms. Thus, heuristics do not tell us anything about the underlying, more proximate, organism-specific and cognitive mechanisms that play a role in perceptual awareness and salience.

The reason we highlighted von Uexküll’s (1957) comparative work is that it provides a window into organism-*specific* rather than organism-general environments. Most of the commentators—Gigerenzer, Noble, Nordli et al., and Schwartz—indeed seem to agree that Uexküll’s idea of a species’ *Umwelt* is a useful way of thinking about these organism-specific factors that shape perception. On the surface, this idea of *Umwelt* may even seem congruent with something like ecological rationality, or perhaps even situational construal (Funder, 2016).

However, in our original article we neglected to discuss von Uexküll’s specific mechanism that directs perception (cf. Noble, 2017)—the type of mechanism that is missing in both the situation-construal and the heuristics and ecological rationality literatures. That is, the way that organisms attend to their unique surroundings or *Umwelt* is guided by species-specific *Suchbilder*—that is, a representation or schema of what is being looked for and what might be selected as the answer or solution. It is this *Suchbild* that directs perception toward the awareness of, generation, and finding of certain cues, such as relevant objects or, say, sources of food. For example, many species of frog will not recognize a fly directly in front of them unless it moves.

All-seeing models of rationality abstract away from these specifics, preferring to deal with cues themselves or various types of universal search (e.g., Gershman, Horvitz, & Tenenbaum, 2015). Gigerenzer et al.’s emphasis on “cross-species *commonalities*, “organism-*general* factors,” and taken-for-granted cues makes precisely this point. The idea of heuristics is so interwoven with cues themselves (Gigerenzer & Gaissmaier, 2011) that the directedness of perception is sidelined (see the next section for further discussion). The various universal or general search rules—for example, “search through cues in order of their validity,” “search through cues in predetermined order,” “search through cues in any order”—don’t provide us with any form of specificity. Even the idea that “search rules specify in what direction the search extends in the search space” doesn’t make sense without some kind of problem, question, or *Suchbild*. It is worth noting that we do not object to the term “heuristic” per se. Rather, we think that alternative conceptualizations of heuristics could be conducive to the idea of *Suchbild*, which could thereby resolve the all-seeing-eye problem. For example, Michael Polanyi uses a *Suchbild*-friendly notion of heuristic when he argues that “the simplest heuristic effort is to search *for* an object you have mislaid”—in other words, as was discussed in our original article, we attend to the world with some form of directed “readiness to perceive” (Polanyi, 1957, pp. 89, 94). Organisms similarly approach situations and visual scenes with some kind of problem or question in mind, and an associated image of the potential answer or solution, which then directs our observation and perception. We next turn our attention to this issue.

***What is the question?***

Our central argument in the original article is that perception is *directed*, and that this matters for our understanding of mind, cognition, and rationality. Perception does not make sense without a focus on directedness. Any scene, situation, or environment features an infinite variety of cues, stimuli, and potential facts. Organisms “cut through” all this potential clutter by directing themselves toward the cues that are relevant to a specific problem at hand, or put differently, they attend to those cues that are relevant for answering particular questions. Some cues are important and relevant for some purposes, and those very cues might be irrelevant for other purposes—depending on the question. Our commentators might largely agree with this. But as we discuss below, the theories that they point toward do not address the directedness of perception—that is, the mechanisms behind perceptual awareness and salience. To speak of any form of rationality and cognition requires that we offer a theoretical approach to this directedness, rather than placing our emphasis on cues or heuristics themselves.

Cues are consequences of questions, problems, and theories, which drive cue salience and awareness. To illustrate this idea, Karl Popper once conducted a playful thought experiment during a public lecture. He asked his audience to simply “observe” their surroundings:

My experiment consists of asking you to *observe*, here and now. I hope you are all cooperating and observing! However, I feel that at least some of you, instead of observing, will feel a strong urge to ask: “WHAT do you want me to observe?” For what I am trying to illustrate is that, in order to observe, we must have in mind a definite question, which we might be able to decide by observation. (Popper, 1967, p. 259, emphasis in the original)

Notice how Popper’s lecture hall is much like Funder’s social situation (cf. Funder, 2016; Rauthmann et al., 2015) where we can presumably, “in principle,” capture what is around us: a lecture hall, fellow audience members, curtains, a pulpit, and any number of other facts. Observers could respond to a battery of questions and report what the situation is about. But latent and potential cues abound and surpass any ability (or reasonable desire) to fully describe them. For example, we could capture any number of observations about any number of the attendees, perhaps survey them to see what they observed. Even saying that the situation is *largely* about this or that is problematic. As Popper notes, there is no pure perception or observation, since observation is always theory-laden: “observation comes after expectation or hypothesis.” In other words, “we learn only from our hypotheses what kind of observations we ought to make: whereto we ought to direct our attention: wherein to take interest” (p. 346). The world is neutral. But there is no neutral observation of the world.

Without the questions and problems that organisms bring to tasks, a focus on cues and environments makes no sense. Organisms always attend to scenes and situations actively looking *for* something—rather than passively recording or absorbing stimuli. We come to encounters with reality with something in mind: with expectations, hypotheses, questions, and theories. This is the human equivalent of the aforementioned *Suchbild*. Focusing on questions and the directedness of perception makes the notion of attention superfluous.

One way to illustrate the importance of problems and questions—and their relevance in directing perception in situations and environments—is to think about this process as a form of forensic problem solving and investigation (cf. Koenderink, 2012). A thought experiment might help make the point. Imagine that someone is found murdered in the aforementioned lecture hall, directly after the lecture, just as audience members are getting up from their seats and exiting the venue. This represents a situation in which all manner of environmental cues could be gathered as evidence for what happened and who the culprit might be. We could begin the analysis by listing and capturing all the objective facts on the scene: people and motives, interviews of audience members (reporting sundry observational fragments), security camera footage or photographs, seating charts or arrangements, visits to the restroom, bank accounts, relationships, smoldering cigar butts outside the back entrance, wine stains on desks, crumpled papers with scribbles, incoming and outgoing text messages, and overwhelming amounts of microfibers and potential DNA evidence—in short, an infinitely structured environment and landscape. The problem is that there is no end to what could be captured, but more importantly, there is no end to what might be relevant and counted as evidence. (Incidentally, just as there is no end to the facts that might be relevant in the gorilla scene.) Much of this amassing of cues, observations, facts, and evidence would involve within-situation and scene-related data and facts. But the scope of potential cues and observations would undoubtedly also include a wild array of facts and observations external to this particular scene, situation, and environment. For example, interviews of audience members may yield the observation that a particular pair of individuals were seen—corroborated by a number of people (Funder’s competent observers: whether *in situ* or *ex situ*: Rauthmann et al., 2015)—to be interacting during the lecture break, and that these individuals were also seen together by several witnesses at a café the day prior to the murder, observed by one witness to be whispering to each other. These are objective facts. But they are just data and facts—perhaps relevant to the case, perhaps not.

The problem is that all of these objective cues and facts do not themselves actually tell us anything. (Note that the same goes for the Simons–Chabris, 1999, gorilla experiment and scene that we discussed in our original article.) The question is: How do we process this or any other situation or scene? Cues and facts inherently do not somehow signal their meaning and relevance in any way. There is no obviousness. And the objective cues and facts that are present could tell us any number of things. Furthermore, any number of cues could objectively be amassed to point to any number of audience members as the culprit. Anything could be relevant.

Now consider how this hypothetical scenario and situation might be approached from a perspective of ecological rationality. The theory, after all, is about evaluation, processing, and rationality, and as was reiterated in two of the commentaries (Gigerenzer, 2017; Nordli et al., 2017), about judgment and decision making under uncertainty. The problem is that we are immediately stuck with needing to define the concept of cues. In our hypothetical scenario the potential cues and facts are innumerable, as Gigerenzer and colleagues would surely agree. But the valid, right, redundant, or weighted cues don’t somehow emerge and array themselves, even though that is precisely what heuristics are supposed to do. And if we look at the “content” of heuristics, they provide no further guidance for how to process the scene and situation. Heuristics are *by definition* constituted by various generic search rules for cues (see the discussion of “search through cues”: Gigerenzer & Gaissmaier, 2011). In other words, the literature assumes that organisms somehow go straight to the correct cues, and that they “search through cues in order of their validity” (Gigerenzer & Brighton, 2009; Gigerenzer & Gaissmaier, 2011; Gigerenzer & Todd, 1999) or “examine cues in the order of their accuracy” (Luan et al., 2014). But saying that we should “search through cues in order of their validity” begs the question of what the relevant cues might be.^3^ Uncertainty is presumed to be reduced through cue-related factors such as “cue redundancy and correlation, the number of observations and cues, and cue weights” (Gigerenzer & Gaissmaier, 2011, p. 457). But again, none of the particular heuristics—whether the gaze heuristic or the recognition heuristic—offers guidance about salience or awareness, because each takes cues (and their weights, numbers, and comparisons) as a given.

Our view is that *questions*—not the nature or structure of cues, or even heuristics—reduce uncertainty and tell us what to direct our perception and observations toward. The mechanism that allows an organism to arrive at cues is a *Suchbild*-like question, not a heuristic that automatically arrays or searches through cues. This distinction is critical. It fundamentally changes the nature of the problem from one of having to deal with overwhelming amounts of information and stimuli—the so-called *problem of attention*—to one in which questions direct awareness toward what even might count as a cue (or “clue”), and what is salient and relevant. The emphasis on questions also changes the perceptual problem from one focused on attention to one focused on awareness, a central distinction.

To return to our thought experiment in the lecture hall, we propose that this situation is best approached with a set top-down guesses, expectations, conjectures, hypotheses, and theories that we impose on it. A prototypical detective like Sherlock Holmes operates in this fashion. Rather than amass reams of observational detail, facts and seeming evidence, gathering everything in sight—an impossible task—the investigation starts with some kind of hypothetical plot. Initial hunches, observational scraps, and hypotheses about who the culprit might be direct the observations of the detective to those cues (or clues) that might be relevant. As suggested by Arthur Conan Doyle’s Sherlock, for others the problem “lay in the fact of there being too much evidence”—that is, any number of things could constitute relevant evidence. But Sherlock operates with a type of *Suchbild* that creates salience and helps generate relevant cues—that is, questions direct his observation to those cues or facts that are relevant to the situation at hand. Note that this is not done on the basis of any kind of algorithm that tallies, weights, or counts cues in some fashion (Gigerenzer & Gaissmaier, 2011). There is no generic “Sherlock heuristic” for arraying or processing cues. Of course, the method of posing questions and problems, and letting conjectures and hypotheses drive observation and perception is not infallible. In our hypothetical situation, investigators will undoubtedly be led down a number of blind allies and focus attention on irrelevant facts. But the cognitive or organism-specific factors are what drive this activity—and subsequent revisions of the underlying theory lead to success. We see this type of forensic investigation as a powerful metaphor for the more general problem of explaining perception, cognition, and rationality as well. 4

Note the parallel between our hypothetical thought experiment and the gorilla experiment (Simons & Chabris, 1999). Our central point is that with any visual scene or environment we are always looking *for* something, and therefore necessarily ignoring any number of other things that are present. The problem thus—*pace* Gigerenzer and colleagues—is not so much about attention or the amount of potential information, but rather how questions and theories direct our observation to the relevant facts. Thus, even the very idea of trying to characterize environments through various taxonomies—as “hostile” (Stanovich, 2017) or as having “high or low uncertainty” or cue redundancy (Gigerenzer & Gaissmaier, 2011)—doesn’t make sense, because the nature of environments is fundamentally tied to the organisms, *Suchbilder*, problems, and questions at hand. From our perspective, the central matters related to perception, cognition, and rationality have to do with the organism and the question, rather than the environment. A *Suchbild* provides the question (how many basketball passes?), which in turn serves as the prime toward the relevant cues. If we are primed to count basketball passes, we’ll attend to these, at the “expense” of other factors. But to say that missing the gorilla illustrates that the human mind is “blind to the obvious” (Kahneman, 2011, pp. 23–24)—or even the idea that visual scenes or situations can “in principle” be exhaustively or even partially represented (Funder)—misspecifies the nature of both perception and reality. All-seeing ideas miss the directed nature of perception and the multifarious nature of reality and how problems, questions, and theories direct our awareness and selection of particular cues. Any visual scene is infinitely structured, and thus only with plots and questions do facts look obvious, particularly in retrospect. And any fact, of course—depending on the question at hand—can have multiple meanings and relevances.^5^

***Equilibria, $500 bills, and the axioms of rationality***

The final set of comments focus on the nature of equilibria, as they relate to perception and the all-seeing-eye argument, and the idea of the axioms of rationality. We discuss each in turn.

In their commentary, Chater and Oaksford argue that our all-seeing-eye argument may have some limited application to rational “functionalist” explanation, but that our arguments do not apply to equilibrium-based analysis and explanations in economics (and related domains). We find Chater and Oaksford’s claim surprising, because the very premise of the cognitive and psychological critique of economics (Kahneman, 2003; Simon, 1978; for a review, see Thaler, 2016) is that equilibrium analysis assumes agent omniscience and rationality (cf. Buchanan, 1959; Kirman, 1992)—an all-seeing eye. But given Chater and Oaksford’s commentary, clearly some further discussion is needed. It is important for us to show that arguments about the nature of *perception* and *observation* are not just tangentially related to equilibrium analysis, but foundational to the theoretical, empirical, and mathematical framework of economics.

The strong assumption behind equilibrium analysis is that any unique information is already priced into assets, stocks, or resources, and thus there are no meaningful arbitrage opportunities. Markets are assumed to be efficient. The observations of many profit-maximizing/seeking agents remove any possibility of finding value. Economists frequently make this point by using the example of a hypothetical $500 bill on the sidewalk: “there are no $500 bills on the sidewalk; if there had been, someone would have already taken them” (Akerlof & Yellen, 1985, pp. 708–709; see also Frank & Bernanke, 2003). If agents happen to (randomly) stumble onto any opportunities, these are quickly snapped up. What might look like an opportunity should be treated suspiciously, as equilibrium theories assume that opportunities are obvious and thus self-eradicating (Ball, Mankiw, & Romer, 1988; Olson, 1996; cf. Fisman & Sullivan, 2016). This strong version of efficient markets and equilibrium has of course been softened and relaxed, allowing for agents to adapt, search, and learn (e.g., Bray & Savin, 1986; Lucas, 1986), including through the use of Bayesian approaches and models (e.g., Aumann, 1987). But the central assumption remains that any “$500 bill”-type arbitrage opportunities are quickly seen—given learning and enough eyeballs—and competed away. Thus, the essential equilibrium architecture of economics, with some minor amendments, remains firmly intact. It’s this model that Chater and Oaksford point toward.

The equilibrium axiom of economics is a direct theoretical analogue to the perceptual problem we have discussed. The question is whether we can assume that the world (or reality) can—perceptually or observationally—be exhausted and somehow fully described and represented. Equilibrium approaches, just like inverse optics and psychophysics, assume that this is possible. The world is assumed to feature various “obvious” objects and things—whether $500 bills on sidewalks or gorillas in visual scenes—and humans perceptually capture these facts into a full representation of the world. As we discussed above, if subjects don’t see these “obvious” things, they are labeled irrational, blind, bounded, or biased, which provides the underlying (for us problematic) foundation for much of behavioral economics (Kahneman, 2011; Thaler, 2016). From an equilibrium perspective, in its strongest form, the correct, full representation is expected to be held by every agent in godlike fashion (in the form of rational expectations; Sargent, 2015). Alternatively, the full representation is an emergent outcome of lots of agents “eyeing”—scanning proverbial sidewalks for $500 bills—and interacting, and building up an aggregate, exhaustive conception of the world through aggregate information processing (Muth, 1961). The economic equilibrium is the all-seeing eye.

The problem in the economic context, just as in the context of observing visual scenes, situations, or environments, is that no exhaustive representation is possible. The varied possible stimuli and cues don’t signal their own relevance. Economic agents do not encounter a world in which obviousness or salience is somehow built into observations. Put differently, objects of relevance don’t (necessarily) have price tags telling us of their value, just as any given item in our visual scene is meaningless without some mechanism of awareness or salience—without a problem, question or theory. Thus the idea of price—the central construct of equilibrium analysis—can be seen as equivalent to Kahneman’s natural assessment (e.g., “size, distance, and loudness”; Kahneman, 2003, p. 1453), serving a similar function of exhausting or fully capturing reality. Both equilibrium and behavioral approaches assume this all-seeing eye and lack underlying mechanisms to account for heterogeneity and the directedness of perception. Thus, we disagree with Chater and Oaksford’s claim that equilibrium analysis does not feature an all-seeing eye. Instead, equilibrium-based approaches assume that observational obviousness reigns, which exhausts the potential for novelty and alternative uses.

It is also here that Chater and Oaksford’s distinction between functional and equilibrium explanation fails. Equilibrium analysis assumes that the functional uses of assets are fully listable, and that all possible uses of objects are known and, in effect, priced in. This is the equivalent of arguing that visual scenes can be exhaustively described, or that the affordances of objects (e.g., assets) can be fully accounted for (cf. Felin, Kauffman, Koppl, & Longo, 2014). But no camera or computation—nor any number of eyes observing or scanning—can fully capture a scene or situation, nor can it exhaust the set of uses and economic possibilities. The set of possible, functional “affordances” for any object are not listable. The problem of accounting for functional uses and affordances, though the perceptual foundations of this have not been explicitly pointed out, has also been noted across economic and biological environments (Kauffman, 2016; Noble, 2016). This idea is also embedded in the criticisms that others have made about equilibrium analysis. For example, Frydman and Phelps have argued that most equilibrium-based approaches represent “fully predetermined models” that “rule out the autonomous role of expectations” (Frydman & Phelps, 2001, p. 2). Sargent further noted that equilibrium-based approaches “preclude any heterogeneity of beliefs” (Sargent, 2015, p. 18). Finally, Edmund Phelps makes an even stronger claim that “the neoclassical idea of equilibrium has not illuminated how the world economy works” (Phelps, 2007, p. xv). We concur. And our argument is that the perceptual and observational assumptions—similar to inverse optics—behind equilibrium analysis are at fault.

The all-seeing eye is also directly embedded in the mathematics used in equilibrium analysis in economics (see Arrow & Debreu, 1954; Muth, 1961; Samuelson, 1971; Walras, 1954; cf. Romer, 2015). Equilibria are modeled as state or phase spaces, building on similar models in physics (cf. Nolte, 2010). That is, economies or markets are represented with the tools of Euler–Lagrangian or Hamiltonian-type phase spaces, where agents, assets, and their prices interact in a closed system, which can be *exhaustively* captured and computed. What these models represent is a form of Laplacean demon that automatically computes and searches through all possible states and spaces, exhausting and saturating them (cf. Noble, 2017). The market simply, in camera-like fashion, works and computes itself (cf. Coase, 1937). All agents are homogeneous, have the same information, allowing for no heterogeneity. Opportunities are obvious and given—just as reality is obvious and given. But just as we have a concern with exhaustive, all-seeing representations in the context of visual scenes and situations, we have the same concerns relative to equilibrium analysis.

Now, all that said, Chater and Oaksford argue that equilibrium analysis does not assume that agent perceptions necessarily need to match external reality, our all-seeing eye. Specifically, they argue that “no [all-seeing] characterization of the external world is required in order to make sense of rational equilibrium explanation, which depends purely on the coherence of the internal elements of the system” (p. 13). It’s this internality that we find problematic—particularly the exhaustive nature of it that is presumed, along with the strong assumptions that are made about agent omniscience. But beyond this, surely economists and social theorists are concerned about our lack of ability to anticipate and capture things like economic bubbles, which cannot be done with equilibrium approaches (Shiller, 2015).

While Chater and Oaksford advocate equilibrium-based approaches, Stanovich makes broadly related arguments about the “axioms of rationality.” He argues (p. 43) that the issues raised by our all-seeing-eye argument “have been resolved for some time now,” and even suggests that *none* of our arguments about perception have any relevance to the “Great Rationality Debate”—and particularly its synthesis—in cognitive science. To summarize, Stanovich’s rationality synthesis consists of the argument that biases pervade human decision-making and that our evolutionary past has not prepared us for the hostile and lab-like decision situations of the modern world. Stanovich argues that biases are pervasive, but his work also points out that there is variance amongst individuals in terms of their susceptibility to bias and their likelihood of matching the “axioms of rational choice.” Some individuals—due to “analytic intelligence” and “cognitive ability” (which override lazy Type 1 processing)—behave according to the axioms of rational choice. Many do not. This has recently allowed Stanovich and colleagues to develop a so-called “rationality quotient.” Stanovich claims that it is this approach—focused on the deviations from the axioms of rationality—which is “ideally suited to studying cognition in the modern world” (p. 47). We respectfully disagree.

To illustrate the problem we have with Stanovich’s axioms of rationality, consider an experimental setting where subjects play the ultimatum game (Güth, Schmittberger, & Schwarze, 1982). We could say that the rational response for the first player (the proposer) in the ultimatum game is to offer the minimum possible increment (e.g., $1, if handed ten $1 bills by the experimenter), and that the second player’s (the responder) rational response is to accept anything that is offered; even one cent, as they clearly are better off. This is one axiom of rationality. And we could then show how some subjects are rational (as either proposers or responders), and then point to poorly performing subjects—postexperiment—and highlight how they violate the norm of rationality. We could, in effect, secure what Stanovich calls “postexperimental endorsement” for the axiom from the subjects: That is, offers should be as low as possible and responders should accept any offer. But note that the axioms themselves are artificially constructed on the basis of the *ex ante* hypotheses of the scientists, rather than telling us anything meaningful about rationality itself. All manner of axioms can be construed as rational. A deviation from the norm could have any number of reasons. What is rational in this context? For example, in some cultural settings it may be rational to propose splits that are higher than 50% (cf. Henrich, Boyd, Bowles, & Camerer, 2005) and to reject offers that are less than that. And any number of small tweaks to the experiment—blind versus face-to-face interaction, player matching (stranger vs. friend), the type of social interaction or mode of communication that is allowed, single versus repeated games, iterative role reversals (proposer becomes responder)—could lead to wildly different outcomes. Now, perhaps this particular axiom of rationality (in the case of a one-shot ultimatum game: offer low, accept low) is one that Stanovich disagrees with. But this illustrates our point about the problem of defining axioms and norms, since they can vary wildly—reinforcing our original points about the multistability and multifaceted nature of rationality.

As Stanovich points out, this type of discussion of many rationalities—as illustrated by the above ultimatum game—might illustrate his point that we are “Panglossians” (Kahneman, 1981). Panglossian explanations are ones that question axioms of rationality by pointing toward one of the following: random performance errors, computational limitations, incorrect norm application, or alternative problem construal. Here we concede. If it is Panglossian to say that we can come up with any number of alternative explanations (and axioms) for why individuals deviate from scientist-specified norms and answers (e.g., offering or accepting a minimum increment in an ultimatum game), norms and rationality (in essence, an all-seeing eye)—then yes: Panglossians we are. In short, we find the program of specifying axioms and then pointing out deviations to be highly problematic.

What Stanovich seems to have in mind when speaking of the axioms of rationality is focused on varied computational or statistical reasoning tasks, which humans appear to fail routinely (and in which there is significant variance). Our first concern is that making these tasks out to be *the* task of the rationality literature in the cognitive sciences is, from our perspective, extremely problematic (Stanovich, West, & Toplak, 2016, pp. 1–14). But we’ll set that aside (see also Schwartz, 2017). The gist of this program of research is the identification of a host of axioms—see his “comprehensive assessment of rational thinking” (CART); Stanovich, (2016) and Stanovich et al. (2016)—in specific domains such as probabilistic and statistical reasoning, scientific reasoning, probabilistic numeracy, rejection of antiscience attitudes, or rejection of superstitious thinking. And these tests then provide an aggregate score of how rational an individual is. Now, for delimited situations, this could be a worthwhile enterprise. However, many of these tests of reasoning—carefully crafted and calibrated, often worked out by scientists over the years—are loaded and only show that the tests are difficult (Krueger & Funder, 2004), and they all too conveniently align with the bias-focused priors of researchers. They fit the “mistakes are fun” school of psychological research. But at their worst these tests of rationality can be seen as an attempt to ensnare unsuspecting experimental subjects into error, with the tests of rationality providing an arsenal of potential ways through which irrationality can be proven. To further compound the problem, scientists themselves can easily create mental contaminations (attributing these to the subjects)—for example, by priming (as illustrated by the gorilla example)—to prove irrationality, blindness or bias.

Some of the more striking claims that have emerged from this program of research are telling. For example, Stanovich points to the incongruence of human first- and second-order preferences (see also Chater & Oaksford’s argument above) and concludes that “humans are often less rational than bees in an axiomatic sense” (Stanovich, 2013, p. 13). Stanovich clearly has a very specific, all-seeing conception of rationality in mind here. But if we compare humans and animals in this all-seeing sense, then the set of possible ways of showing irrationality indeed is innumerable. We could, for example, compare human and rat maze navigation. We don’t find this useful, nor in any way related to cognition and rationality. Our goal in the original article was to highlight species-specific factors—to understand the nature of the organism—rather than comparing the rationality of, say, bees and humans. We follow Tinbergen, who regarded the power of the comparative method as residing in the focus on species specificity, rather than in trying to “formulate theories claimed to be general” (Tinbergen, 1963, p. 411). Some animals indeed appear to be “rational” at some tasks, and “irrational” at others—though making comparative claims about rationality is only an artifact of the all-seeing standards (and the derivative experiments) set up by scientists, rather than telling us anything of substance about rationality and cognition itself.

What does all of this have to do with perception? After all, Stanovich argues that our article doesn’t say anything important about rationality: “No important conclusions about rational thought depend on issues of perceptual theory at the level dealt with in Felin, Koenderink, and Krueger’s (2017) essay” (p. 42). The points made above, about the problematic nature of the axioms of rationality, hopefully show just how important our arguments about perception are for the domain of rationality. But in terms of Stanovich’s commentary, here the specific link between perception and rationality has an important meta-theoretical component. If scientists begin with the *prior* that human rationality is biased, then this theoretical prior will guide the construction of experimental tests and subsequent observations toward finding this failure and error. Perception and observation are theory-laden. And this goes for both scientists and human subjects.

Stanovich notes in his commentary that “in fact, there is no way to tell whether there has been too much or too little emphasis on bias” (p. 47). We certainly agree. It is unclear, though, how Stanovich then—only a few paragraphs later—can simultaneously claim that “unfortunately, the modern world tends to create situations in which the default values of evolutionarily adapted cognitive systems are not optimal” (p. 48). These two claims are incongruent, and the latter statement is unverifiable. High-level, aggregate improvements in general human welfare, radical technological progress, and any number of other metrics would suggest that Stanovich’s pessimism about the human mind is not warranted. And importantly, it seems that Stanovich’s own theoretical priors—or theoretical *Suchbild*—are driving his claims about rampant bias and his concerns about cognitively “hostile” environments. Much of the nonoptimality that he observes is constructed in labs to suit the a priori expectations of scientists and their theories. Stanovich, in fact, emphatically argues that life “*is becoming more like the tests!*” (p. 48). This is a strong claim. But as Schwartz (2017) discusses in his commentary, “we will never understand [the mind] by creating an environment, like the laboratory, that distorts [its] fundamental nature.” What the lab masks, from our perspective, is that in the real world not only do people self-select into situations, but also (in effect) select into cues and primes (through questions and theories), rather than being randomly assigned into conditions (in which they might be primed or mentally contaminated by factors such as, say, the color of an object or the temperature of the room). In the lab this type of self-selection is purposefully avoided through random assignment, thus confounding the analysis and stacking the deck (through primes and questions) toward rational or irrational outcomes, depending on the scientist’s own theories. Thus, we would argue that many of the experimental findings about rationality scarcely translate into the real world, where people seem to muddle through just fine. Do individuals make mistakes? Absolutely. But many of these mistakes and irrationalities are artificially conjured in the lab, similar to the visual illusions and gorilla experiment that we discussed in our original article. In all, despite the devastatingly “hostile” cognitive environments outlined by Stanovich—and though problems undoubtedly abound—human societies nonetheless seem to be less violent and more prosperous than ever before (cf. Mokyr, 2002; Pinker, 2011).

*Conclusion*

We are thrilled about the opportunity to engage in this debate and interdisciplinary exchange of ideas about the nature of rationality, perception, and cognition. Some of the commentaries are strongly critical of our arguments about perception and rationality, though some aspects of our all-seeing eye argument resonate (Noble, 2017; Schwartz, 2017). Undoubtedly scholars are likely to vehemently disagree on these matters, as is readily evident from the commentaries. From our perspective the commentaries illustrate just how pervasive the all-seeing eye assumption is, beyond the work of Herbert Simon and Daniel Kahneman. The assumption manifests itself in varied ways across psychology and cognitive science—including the situation-construal literature (Funder, 2017), the literature on heuristics and ecological rationality (Gigerenzer, 2017; Nordli et al., 2017), functional and equilibrium analysis (Chater & Oaksford, 2017), and the literature on rationality and the psychology of reasoning (Stanovich, 2017). Beyond these literatures, the all-seeing eye is also the central assumption among many in philosophy (e.g., Block, 2015; Burge, 2010), vision science (e.g., Geisler, 2011; Ma, 2012), computer science (e.g., Gershman et al., 2015), and economics (e.g., Frydman & Phelps, 2001; Muth, 1961; Thaler, 2016). Perceptual assumptions tend to be deeply hidden within most theories, and of course deeply embedded in the very nature of empirical observation and science itself. Thus, we hope that this debate and set of commentaries will open up further discussion and dialogue, which in turn will allow for productive theoretical and empirical investigations to further our understanding of rationality, mind, and cognition across the sciences.

## Footnotes

1 Funder specifies that he is interested in cues at the “middle level of analysis” (Funder, 2016, p. 205)—that is, not concrete details about, say, temperature or the number of people involved in the situation, nor broad categories such as that a situation is, say, a party or meeting—but rather, “experientially salient aspects” of the situation. The problem here, as we will discuss, is that the mechanism behind perceptual salience or awareness is not provided.

2 The pervasive focus on cues is also evident in discussions of specific heuristics as well; for example, in a recent review of the “recognition heuristic,” *cue* is referenced over one hundred times (Goldstein & Gigerenzer, 2002).

3 Interestingly, the distinction between “searching for” versus “searching through” cues is quite important here—though it is informative, and for us problematic, that the emphasis seems to be on the latter (Gigerenzer & Gaissmaier, 2011).

4 Of course, not just forensic investigation, but science itself can be discussed in terms of how questions, hypotheses, and theories drive observation and perception (e.g., Bell, 1990; Polya, 1945; Popper, 1967).

5 We might here conclude and concur with Arthur Conan Doyle’s Sherlock by saying that “there is nothing more deceptive than an obvious fact.” Thus the gorilla (Simons & Chabris, 1999)—“obvious” to Kahneman (2011)—is only obvious if it is relevant to a set of questions and theories.
